# A modified Dung Beetle optimizer for combined heat and power economic dispatch considering power losses, valve-point effects, and operating constraints

**DOI:** 10.1038/s41598-025-32060-4

**Published:** 2026-01-16

**Authors:** Mahmoud Rihan, Mohamed Ebeed, Noor Habib Khan, Raheela Jamal, Mosaed Elnaka, Morsy Nour, Amal M. Abd El Hamid, Hany S. E. Mansour

**Affiliations:** 1 Department of Electrical Engineering, Faculty of Engineering, Qena University, Qena, 83521 Egypt; 2 Electrical Department, Faculty of Engineering, Sohag University, Sohag, 82524 Egypt; 3 Department of Electrical Engineering, University of Jaén, 23700 EPS Linares, Jaén, Spain; 4 College of Mechanical and Electrical Engineering, Qingdao Binhai University, Shandong, 266555 China; 5 Department of Electrical Engineering, Faculty of Energy Engineering, Aswan University, Aswan, 81528 Egypt; 6 Electrical Department, Faculty of Technology and Education, Sohag University, Sohag, 82524 Egypt; 7 Electrical Engineering Department, Faculty of Engineering, Suez Canal University, Ismailia, 41522 Egypt

**Keywords:** Economic dispatch, Modified Dung Beetle optimizer, Prohibited operating zones, Power losses, Valve-point-loading-effect, Energy science and technology, Engineering, Mathematics and computing

## Abstract

The Combined Heat and Power Economic Dispatch (CHPED) problem represents a significant optimization challenge in modern power systems due to its inherent complexity arising from multiple operational constraints. This complexity is further exacerbated when considering the effect of power losses (PLs), valve-point loading effect (VPLE), and prohibited operating zones (POZs). Consequently, an efficient and robust optimization algorithm is essential for obtaining a globally optimal solution while satisfying all constraints. To address these challenges, this work evaluates the effectiveness of the Modified Dung Beetle Optimizer (MDBO) for solving the CHPED problem, considering PLs, VPLE, and POZs. The MDBO enhances the search process and mitigates the limitations of the conventional Dung Beetle Optimizer, particularly stagnation and premature convergence to local optima. The novelty in this paper is proposing a modified version of the traditional DBO (MDBO) by integrating three improvement strategies, including the fitness distance balance (FDB), Chaotic mutation (CM), and adaptive local search approach (ALSA), to solve the CHPED problem. The proposed MDBO has the ability to overcome the shortcomings of traditional DBO, such as its premature convergence and tendency to local optima. The effectiveness of the proposed MDBO has been evaluated on CHPED problems involving 4-unit, 7-unit, 24-unit, and 48-unit systems under various operating conditions. Moreover, MDBO performance has been rigorously assessed using standard benchmark test suites, including CEC-2019. The results demonstrate a significant reduction in operating costs, confirming the superior performance of the MDBO in comparison to existing optimization techniques like Sand Cat Swarm Optimizer (SCSO), African vultures optimization algorithm (AVOA), Sine Cosine Algorithm (SCA), Harris Hawks Optimization (HHO), Grey Wolf Optimizer (GWO), Liver cancer algorithm (LCA), Zebra Optimizer Algorithm (ZOA), and Whale Optimization Algorithm (WOA). Furthermore, the proposed algorithm consistently outperforms alternative methods reported in the literature, offering a more efficient and reliable solution for CHPED optimization.

**Supplementary Information:**

The online version contains supplementary material available at 10.1038/s41598-025-32060-4.

## Introduction

### Background and problem context

Combined Heat and Power (CHP) systems, also known as cogeneration systems, represent a highly efficient and sustainable energy solution applicable across diverse sectors. By producing electrical and thermal energy, CHP systems significantly enhance overall energy utilization and offer substantial environmental advantages. In the context of the global shift toward cleaner and more efficient energy technologies, CHP systems play a pivotal role in reducing greenhouse gas emissions, enhancing energy reliability, and delivering long-term economic benefits. Depending on the technology and its specific application, CHP systems can achieve efficiency levels ranging from 60% to over 80%.

CHP units are consistent with a wide diversity of fuel sources, involving natural gas, biogas, coal, oil, and renewable energy sources such as biomass and solar thermal energy. Unlike conventional power plants that lose a large part of input energy as heat during electricity generation, CHP systems recover and employ this thermal energy to meet heating demands, thereby pushing total system efficiency to nearly 90% ^1,2^. Moreover, CHP systems have the prospect to decrease total fuel costs by 10–40% and lower pollutant emissions by 13–18% ^3^. To completely harness the advantages of CHP systems, it is crucial to address the Combined Heat and Power Economic Dispatch (CHPED) issue, which involves determining the optimum cost-effective schedule for both power and heat generation under various operational constraints ^4^.

## Research gap and motivation

Traditional optimization approaches for solving CHPED include the normal boundary intersection method ^5^, branch and bound ^6^, lagrangian approach ^7,8^, sequential quadratic programming ^9^, and benders method ^10,11^. However, these methods often depend heavily on initial conditions, require numerous iterations, and may not consistently converge to the global optimum. Additionally, they can be mathematically complex and computationally intensive.

As a result, heuristic and metaheuristic algorithms have emerged as powerful alternatives for solving CHPED problems. A comprehensive survey of heuristic methods for CHPED is presented in ^12^. Genetic Algorithm (GA)-based approaches have been widely applied to minimize total generation costs while taking into consideration valve-point-loading effects (VPLEs) and transmission power losses (PLs) ^13^. Enhanced GA variants featuring improved crossover and mutation strategies ^14^, as well as the Non-dominated Sorting Genetic Algorithm II (NSGA-II) ^15^, have demonstrated effectiveness in handling mixed-variable scheduling tasks and integrating energy storage systems. Particle Swarm Optimizer (PSO) has been extensively employed for CHPED ^16^, with modifications such as time-varying acceleration coefficients to improve convergence behavior and prevent premature stagnation. VPLEs are often modeled using sinusoidal components added to the cost function. To address uncertainties in demand and renewable energy availability, PSO has been integrated with Monte Carlo simulations ^17^. Additionally, PSO has been utilized to assess dispatch strategies for reducing fuel consumption in coal-fired CHP plants ^18^.

Hybrid algorithms have been proposed to solve the CHPED problem. A hybrid PSO–NSGA-II approach simultaneously optimized both cost and emissions while incorporating transmission PLs ^19^. However, many of these methods have been validated only on small-scale systems (e.g., those comprising 5 to 7 units), limiting their generalizability. The Manta Ray Foraging Optimization (MRFO) has also been implemented to address generation scheduling in systems integrating wind power and VPLEs, achieving cost reductions of up to 8% across both small-scale (5-unit) and large-scale (96-unit) configurations under varying load conditions ^20^. The Cuckoo Search Optimizer (CSO) has emerged as a viable approach for addressing the CHPED issue ^20–23^. Its primary objective is to reduce overall fuel costs while meeting electricity and heat demands, considering constraints such as VPLEs and transmission PLs. In ^21^, CSO was tested on systems of various sizes, including small (5-unit), medium (24-unit), and large (48-unit) configurations. The 5-unit system was evaluated under three distinct heat and power demand scenarios. Similarly, the study in ^22^ demonstrated CSO’s effectiveness across six different test networks with 4, 5, 7, 11, 24, and 48 units. The methodology proposed in ^23^ was applied to five case studies, three involving quadratic cost functions without PLs, and two with non-convex cost functions. Several other metaheuristic approaches have also been employed to solve the CHPED problem, including the grey wolf optimizer (GWO) ^24^, heap-based optimization ^2^, group search optimization ^25^, gravitational search algorithm ^26^, marine predators algorithm ^27^, kho-kho optimization ^28^, a hybrid of weighted vertices-based approach and PSO ^29^. A mixed integer model was proposed to solve the CHPED for 4 units, 5-units, 24-unit, 48-unit, and 96 unit systems ^30^. An improved version of artificial rabbits optimization based on fitness distance balance was presented for solving the CHPED of 4-unit, 5-unit, 7-unit, 24-unit, 48-unit, 96-unit, and 192-unit systems ^31^. The artificial hummingbird algorithm based integrates a linear controlling strategy was developed to solve CHPED for different systems ^32^. An improved version of AVOA based orthogonal oppositional methodology was proposed to solve the CHPED for 48-unit system^33^. A hybrid optimizer based on Harris hawk’s optimizer (HHO), and imperialist competitive algorithm (ICA) was employed to solve the CHPED of 48-unit, 84-unit, and 96-unit systems ^34^.

It is worth mentioning here that metaheuristic optimization algorithms can solve several optimization problems where in a hybrid particle swarm optimizer was presented for optimal coordination of overcurrent relay ^35^ and it can be also applied for optimal placement of electric vehicle stations in distbution grids ^36^. A hybrid simulated annealing (SA) with particle swarm optimization (PSO) was utilized to control variable frequency drives^37^. Artificial intelligence (AI) was proposed for improving the accuracy of objective and physical pressure ratings ^38^. A fast sparse Bayesian learning algorithm was developed for superresolution and the coherent processing of multi-band radar data ^38^. In ^39^, a deep genetic algorithm was suggested for optimizing the channel for signal integrity. In ^40^, a Bayesian posterior loss function technique and a heuristic frequentist approach technique was proposed for to enhance the human decision making.

DBO a robust optimization algorithm that has been applied to solve different optimization problems ^41^. However, several modified versions of DBO were proposed to overcome the stagnation of the traditional DBO to local optima, premature convergence and its low performance where in ^42^, a modified DBO was presented based on Mean Differential Variation and Latin hypercube sampling. A multi-strategy was integrated to DBO based on Levy distribution, two separate cross operators, and Beta distribution to solve path planning problem ^43^. A t-distribution variation approach was applied to enhance the searching ability of the DBO to solve different optimization problems ^44^. Three improvement strategies were integrated to DBO to improve its performance including T-distribution mutation, differential evolutionary variation, cooperative search mechanism and cubic chaotic mapping ^45^. The fractional order calculus methodology was applied with DBO to boost its searching performance in image segmentation ^46^. At the end of this context, the performance of the DBO should be improved for solving large scale problems. Thus, in this paper, three improvement strategies were integrated into DBO to solve the CHPED problem including the fitness distance balance (FDB), Chaotic mutation (CM), and adaptive local search approach (ALSA).

### The key contributions

From the previous review, the main associated gap of the applied methods for solving the CHPED is that some papers solved CHPED without considering VPLEs while some papers solved the CHPED with considering PLs only, and some papers solved the CHPED with considering the POZs only. The aim of the papers is to fill the research gap by solving the CHPED of small, medium and large-scale systems considering VPLEs, PLs, and POZs. In addition to that, a modified optimizer is presented to overcome the shortage of traditional DBO via integration of three improvements including ALSA, CM and FDB. Therefore, the contributions to this study can be depicted as follows:

- New Modified Dung Beetle Optimizer (MDBO) is proposed to solve the CHPED based on boosting the exploitation and exploration of the conventional DBO using three strategies including ALSA, CM and FDB.
- MDBO performance has been rigorously assessed using standard benchmark test and CEC-2019 suites to verify its efficiency and robust performance.
- Solving the CHPED problem, considering PLs, VPLE, and POZs for 4 different test systems and the results obtained were compared with other optimization algorithms including SCSA, AVOA, SCA, HHO, GWO, LCA, ZOA, and WOA.

This article is structured as follows: Section "Problem Formulation" outlines the features of CHP units and formulates the economic dispatch problem using DBO and MDBO, considering VPLEs, PLs, and POZs. Section "Methodology" describes the test systems used for validation, followed by optimization outcomes presented in Section "Numerical Results". A comprehensive evaluation of the suggested approach is provided in Section "Conclusions", and the conclusions are summarized in Sect. 6.

## Problem formulation

The objective of the CHPED problem is to achieve the lowest possible overall operational cost by optimally coordinating three categories of generation units: those producing only electricity, those generating solely heat, and CHP units. To evaluate different solutions, a fitness function is constructed, taking into account the amount of heat and power generated by each unit. The aggregate operational cost is then determined by summing the individual expenses associated with each type of generation unit, as outlined below:

1 $$Cost_{CHP} = \mathop \sum \limits_{i = 1}^{{N_{p} }} Cost\user2{ }_{i}^{P} \left( {P_{i}^{p} } \right) + \mathop \sum \limits_{j = 1}^{{N_{u} }} Cost\user2{ }_{j}^{H} \left( {H_{j}^{u} } \right) + \mathop \sum \limits_{k = 1}^{{N_{f} }} Cost\user2{ }_{k}^{CHP} \left( {P_{k}^{f} ,H_{k}^{f} } \right)$$

where, ${N}_{p}$ refers to the units dedicated solely to electricity generation, ${N}_{u}$ signifies those units that produce only heat, and ${N}_{f}$ indicates the CHP units. The cost terms ${\text{cost }}_{i}^{P}$, ${\text{cost }}_{j}^{H}$, and ${\text{cost }}_{k}^{CHP}$ represent the cost of generation cost of ${i}^{th}$ power-only units, the ${j}^{th}$ heat-only unit, and the ${k}^{th}$ CHP unit. The cost functions for these units are expressed as follows:

2 $${Cost }_{i}^{P}\left({P}_{i}^{p}\right)={A}_{i}{\left({P}_{i}^{p}\right)}^{2}+{B}_{i}{P}_{i}^{p}+{Q}_{i}+\left|{T}_{i}\mathrm{sin}\left({G}_{i}\left({P}_{i}^{p,Min}-{P}_{i}^{p}\right)\right)\right|$$

3 $${Cost }_{j}^{H}\left({H}_{j}^{u}\right)={A}_{j}{\left({H}_{j}^{u}\right)}^{2}+{B}_{j}{H}_{j}^{u}+{Q}_{j}$$

4 $${Cost }_{k}^{CHP}\left({P}_{k}^{f},{H}_{k}^{f}\right)={A}_{k}{\left({P}_{k}^{f}\right)}^{2}+{B}_{k}{P}_{k}^{f}+{Q}_{k}+{T}_{k}{\left({H}_{k}^{f}\right)}^{2}+{G}_{k}{H}_{k}^{f}+{V}_{k}{P}_{k}^{f}{H}_{k}^{f}$$

- For power-only units, cost was modeled using coefficients ${A}_{i}, {B}_{i}$, ${Q}_{i}, {T}_{i},$ and ${G}_{i}$, representing the cost parameters for ${i}^{th}$ electricity production units.
- For heat-only units, cost was modeled using coefficients ${A}_{j}, {B}_{j}$, and ${Q}_{j}$, which account for the heat generation expenses.
- For CHP units, cost was represented using coefficients ${A}_{k}, {B}_{k}{, Q}_{k}, {T}_{k}, {G}_{k}$, and ${V}_{k}$​, which captures the combined cost of electricity and heat production for the ${k}^{th}$ units ^47^.

### Constraints in the CHPED problem

The CHPED problem formulation includes both equality and inequality constraints to ensure energy balance and adherence to operational limits:

#### Equality constraints

The equality constraints include the power balanced with and without power losses asdescribed in Eqs. (5) and (6), as well as the heat balanced of hsystem as decribed in (7). In addation to that the equality constraints include the capacities of only-power units, heat-only units and the CHP units as depicted in (8), (9), and (10), respectivly. Furthermore, the prohibted zones of the generation units are described in (11).

5 $$\mathop \sum \limits_{i = 1}^{{N_{p} }} P_{i}^{p} + \mathop \sum \limits_{k = 1}^{{N_{f} }} P_{k}^{f} - P_{{\text{demand }}} = 0$$

6 $$\mathop \sum \limits_{i = 1}^{{N_{p} }} P_{i}^{p} + \mathop \sum \limits_{k = 1}^{{N_{f} }} P_{k}^{N} - P_{{\text{demand }}} - P_{{\text{losses }}} = 0$$

where; *P*_*losses*_ is power losses, while *P*_*demand*_ is the power demand.

7 $$\mathop \sum \limits_{k = 1}^{{N_{f} }} H_{k}^{f} + \mathop \sum \limits_{j = 1}^{{N_{u} }} H_{j}^{u} - H_{{\text{demand }}} = 0$$

8 $$P_{power\_only} = \mathop \sum \limits_{i = 1}^{{N_{p} }} \left[ {Max\left( {P_{i}^{p} - P_{i}^{pMax} ,0} \right) + Max\left( {P_{i}^{pMin} - P_{i}^{p} ,0} \right)} \right]$$

9 $$P_{heat\_only} = \mathop \sum \limits_{j = 1}^{{N_{u} }} \left[ {Max\left( {H_{j}^{u} - H_{j}^{u, Max } ,0} \right) + Max\left( {H_{j}^{u,Min} - H_{j}^{u} ,0} \right)} \right]$$

10 $$P_{CHP} = \mathop \sum \limits_{k - 1}^{{N_{f} }} \left[ {\begin{array}{*{20}c} {Max\left( {P_{k}^{f} - P_{k}^{f,Max} \left( {H_{k}^{f} } \right),0} \right) + Max\left( {P_{k}^{f,Min} \left( {H_{k}^{f} } \right) - P_{k}^{f} ,0} \right) + } \\ {Max\left( {H_{k}^{f} - H_{k}^{f,Max} \left( {P_{k}^{f} } \right),0} \right) + Max\left( {H_{k}^{fMin} \left( {P_{k}^{f} } \right) - H_{k}^{f} ,0} \right)} \\ \end{array} } \right]$$

11 $$P_{{POz_{s} }} = \mathop \sum \limits_{i = 1}^{{N_{p} }} \left[ {\xi_{i} \mathop \sum \limits_{V = 1}^{{Z_{i} }} Min\left( {Max\left( {P_{i}^{p} - P_{i,V}^{L} ,0} \right),Max\left( {P_{i,V}^{U} - P_{i}^{p} ,\;0} \right)} \right)} \right]$$

#### The inequality constraints

Each power-only unit must operate within its designated generation limits. These constraints ensure that the power output from the ${i}^{th}$ unit stays within the allowable lower and upper bounds.

12 $$P_{{i{\mathrm{,Min}}}}^{{power\_only_{ } }} \le P_{i}^{power\_only} \le P_{i,Max}^{{power\_only_{ } }} i = 1, \ldots ,N_{p}$$

The heat output from each heat-only unit is restricted by its minimum and maximum generation capabilities.

13 $$\left({H}_{j}^{f}\right)\le {H}_{j}^{f}\le \left({H}_{j}^{f}\right)j, {\forall }_{j}$$

where ${H}_{j}^{{f}_{Max}}$ and ${H}_{j}^{{f}_{Min}}$ represent the upper and lower restrictions of heat output from the ${j}^{th}$ unit, correspondingly.

The CHP units concurrently generate both heat and power, are also subject to operational limits.

14 $${H}_{j}^{{f}_{Min}}\left({P}_{j}^{f}\right)\le {H}_{j}^{f}\le {H}_{j}^{{f}_{Max}}\left({P}_{j}^{f}\right)j=1,\dots ,{N}_{f}$$

15 $${H}_{k}^{{u}_{Min}}\le {H}_{k}^{u}\le {H}_{k}^{{u}_{Max}}k=1,\dots ,{N}_{u}$$

The electrical power generation from the ${k}^{th}$ CHP unit must lie within the bounds ${P}_{j}^{{f}_{\text{Max }}}$ and ${P}_{j}^{{f}_{\text{Min }}}{H}_{k}^{f}$, which denote the lower and upper bounds of the power output capacity for the respective unit, respectively. Likewise, the heat output from the same CHP unit must fall within the limits ${H}_{j}^{{f}_{Min}} {P}_{k}^{f}$ ${H}_{j}^{{f}_{Max}} {P}_{k}^{c}$, which denote the minimum and maximum allowable heat generation from the ${k}^{th}$ CHP unit, respectively.Also, Certain regions within the power generation range are deemed infeasible due to operational constraints or safety considerations; these segments are referred to as POZs. The feasible power output range for the ${i}^{th}$ power-only unit is characterized in accordance with Eq. (16). Here, ${P}_{i}^{n,L}$ and ${P}_{i}^{n,U}$ represent the lower and upper limits of the ${n}^{th}$ POZ for the ${i}^{th}$ power-only unit, respectively, while ${Z}_{i}$ denotes the total count of such POZs associated with the unit.

16 $$\left\{\begin{array}{c}{P}_{i}^{\text{ Min }}\le {P}_{i}^{power\_only}\le {P}_{i}^{Max}\\ {P}_{i}^{n,l}\le {P}_{i}^{powe{r}_{only}}\le {P}_{i}^{n,U}, {\forall }_{n} \in \left[\mathrm{1,2},\dots ,{Z}_{i}\right]\\ {P}_{i}^{{Z}_{1},U}\le {P}_{i}^{p}\le {P}_{i}^{p,Max}\end{array}\right.$$

## Methodology

### Dung beetle optimization (DBO)

The Dung Beetle Optimization (DBO) algorithm is a biologically inspired based metaheuristic that simulates the unique behaviors of the dung beetles. It incorporates four key activities: Rolling, Spawning, Foraging, and Stealing, which collectively guide the population of virtual dung beetles searching for optimal solutions.

#### Rollerball Dung Beetle (rolling)

Dung beetles roll dung balls in straight paths while navigating using sunlight. This behavior is modeled in DBO for updating the beetle’s position using Eq. (22) ^41^:

17 $$\begin{array}{c}{x}_{i}(tt+1)={x}_{i}(tt)+\alpha \cdot k\cdot {x}_{i}(tt-1)+b\cdot \Delta x\\ \Delta x=\left|{x}_{i}(tt)-{X}^{w}\right|\end{array}$$

where: ${x}_{i}(tt)$ is the position of the *i*^*th*^ beetle at time $(tt)$. $\alpha$ determines if the beetle deviates from its original path (random directional factor, randomly set to 1 or -1). $k$ is a deflection coefficient (between 0 and 0.2). $b$ is a constant in the range [0, 1]. $\Delta x$ simulates the beetle’s displacement toward the light source. If the beetle encounters an obstacle, it simulates directional change via a dancing behavior, modifying its position based on Eq. (23).

18 $${x}_{i}(tt+1)={x}_{i}(tt)+\mathrm{tan}(\theta )\left|{x}_{i}(tt)-{x}_{i}(tt-1)\right|$$

The angle *θ ⊆ *[0*, π*] determines direction adjustment, with no update occurring when *θ* = 0, *π* ∕ 2, and *π*.

#### Spawning behavior

Dung beetles choose safe zones for laying eggs. DBO models this through adaptive boundaries for local exploration:

19 $$L{b}^{*}=max\left({X}^{*}\times (1-R),Lb\right)$$

20 $$U{b}^{*}=min\left({X}^{*}\times (1+R),Ub\right)$$

21 $$R=1-\frac{t}{{T}_{max}}$$

where, $L{b}^{*}$ and $U{b}^{*}$ is the lower and upper constraints of the spawning region, $Lb$ and $Ub$ is the Original lower and upper constraints, ${X}^{*}$ is the current best solution, $R$ is the decay factor over iterations, while ${T}_{max}$ is the maximum number of iterations.

The position update in spawning is defined by Eq. (22), using random values $b1$ and $b2$.

22 $$Xi(tt + 1) ={X}^{*} + b1 \times (Xi(tt) - L{b}^{*}) + b2 \times (Xi(tt) - U{b}^{*})$$

#### Foraging

Foraging simulates a global search for optimal solutions, influenced by the best global position, modeled as:

23 $$L{b}^{b}=max\left({X}^{b}\times (1-R),Lb\right)$$

24 $$U{b}^{b}=min\left({X}^{b}\times (1+R),Ub\right)$$

where ${X}^{b}$ is the best global position. The position update for foraging by:

25 $${x}_{i}\left(tt+1\right)=Xi(tt)+{c}_{1}\times \left(x\left(tt\right)-L{b}^{b}\right)+{c}_{2}\times ({x}_{i}\left(tt\right)-U{b}^{b})$$

where, ${c}_{1}$ is the normally distributed random number, and ${c}_{2}$ : random vector within [0, 1].

#### Stealing behavior

Some beetles exhibit kleptoparasitic behavior by stealing dung balls from others. This behavior is analogous to the exploitation process in optimization, where certain individuals mimic others to enhance convergence toward promising regions in the search space. In the algorithm, this behavior is modeled using the global best location ${X}^{b}$. The location update for a stealing beetle is given by:

26 $${x}_{i}\left(tt+1\right)={X}^{b}+S\times g\times \left(|{x}_{i}\left(tt\right)-{X}^{*}|\right)+(|{X}_{i}\left(tt\right)-{X}^{b}|)$$

where $S$ is a constant (0.5), $g$ is a random variable and ${X}^{*}$ is the best position globally.

### Modified Dung Beetle optimizer (MDBO) algorithm

To enhance the standard DBO’s performance, the proposed MDBO incorporates the following three key improvements:

#### Fitness distance balance (FDB)

FDB is designed to improve global exploration ^48–56^. In this approach, the populations positions are updated based on a score vector derived from their objective function and the distance between each population and the best solution. The score vector can be determined based on the normalized distance $\mathrm{norm}\left({DS}_{i}\right)$ and fitness values $\mathrm{norm}\left({F}_{i}\right)$ as follows:

27 $${D}_{i}=\sqrt{{\left({X}_{i}^{1}-{X}_{1}^{b}\right)}^{2}+{\left({X}_{i}^{2}-{X}_{2}^{b}\right)}^{2}+\cdots +{\left({X}_{i}^{d}-{X}_{3}^{b}\right)}^{2}}$$

28 $$F=\left[{F}_{1},{F}_{2},\cdots ,{F}_{n}\right]$$

29 $$D=\left[{D}_{1},{D}_{2},\cdots ,{D}_{n}\right]$$

where $\mathrm{D}$ and $\mathrm{F}$ represent the distance and the fitness vectors.

30 $$norm\left({DS}_{i}\right)=\frac{{D}_{i}-{D}_{min}}{{DS}_{max}-{DS}_{min}}$$

31 $$\begin{array}{c}\\ norm\left({F}_{i}\right)=\frac{{F}_{i}-{F}_{min}}{{F}_{max}-{F}_{min}}\end{array}$$

$$min$$ and $$max$$ are subscript denote the lower and maximum values, respectively. The score of each population ($${Sr}_{i})$$ can be obtained as follows:

32 $${Sr}_{i}=\varepsilon \times \left(1-norm\left({F}_{i}\right)\right)+(1-\varepsilon )\times norm\left({D}_{i}\right)$$

33 $$\varepsilon =0.5\times \left(1+\frac{t}{{t}_{max}}\right)$$

where, ${t}_{max}$ represents the maximum number of iterations permitted, while t denotes the iteration count currently being executed.

#### Chaotic mutation (CM)

Introduces chaos-based variation to prevent premature convergence and helps escape local optima, specifically in later phases of the search. The chaotic mutation (CM) mechanism enhances the exploration capability of the proposed optimizer by assigning new solution positions based on principles from chaos theory ^57^. Specifically, the logistic chaotic map is employed for this mutation process, as defined in ^58,59^:

34 $${\tau }{\prime}=\mu \tau (1-\tau )$$

35 $${X}_{i}(t+1)={X}_{i}(tt)+{\tau }{\prime}\times \, \left({U}_{b}-{L}_{b}\right)$$

where $\tau^{\prime }$ refers to the logistic chaotic paramter in which $\tau^{\prime } \ne \left\{ {0.0, 0.25, 0.75, 0.5, 1.0} \right\}$. $\tau$ represents random paramter between 0 and 1. $\mu$ equals to 4.

#### Adaptive local search approach (ALSA)

The Adaptive Local Search Algorithm (ALSA) is designed to improve the local exploitation capability by updating each agent’s position relative to the current best solution. During the iteration process, the population members are adjusted according to:

36 $${X}_{i}\left(tt+1\right)={X}^{b}\left(1-\frac{t}{{t}_{max}}\right)+\left(mean(X\left(tt\right))- {X}^{b}\right)\times rand$$

where $rand$ is a random value in the range of [0,1].

**Fig. 1:**
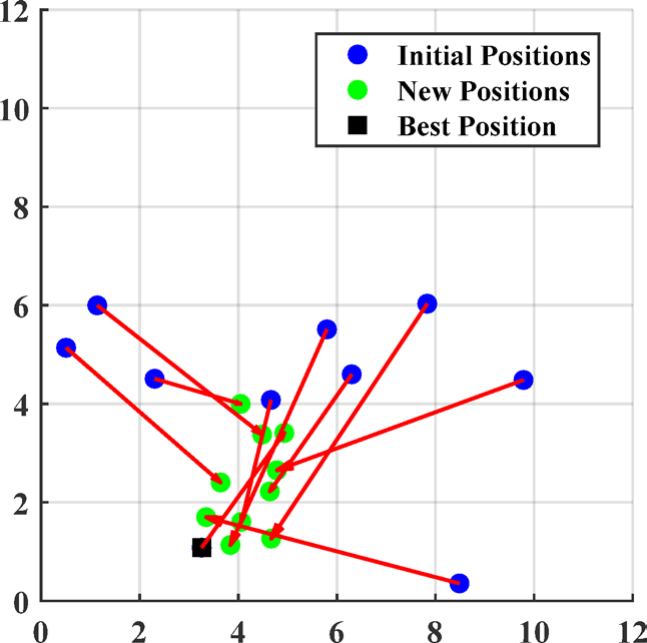
The updated populations based on (ALSA).

This adaptive mechanism ensures that agents remain locally focused on the best-known position while retaining randomness for exploration. Figure 1 illustrates the population update mechanism based on ALSA. A pseudo code the proposed MDBO is depicted in algorithm 1.

The pseudo code of the proposed MDO is presented in Algorithm1.

**Algorithm 1:**
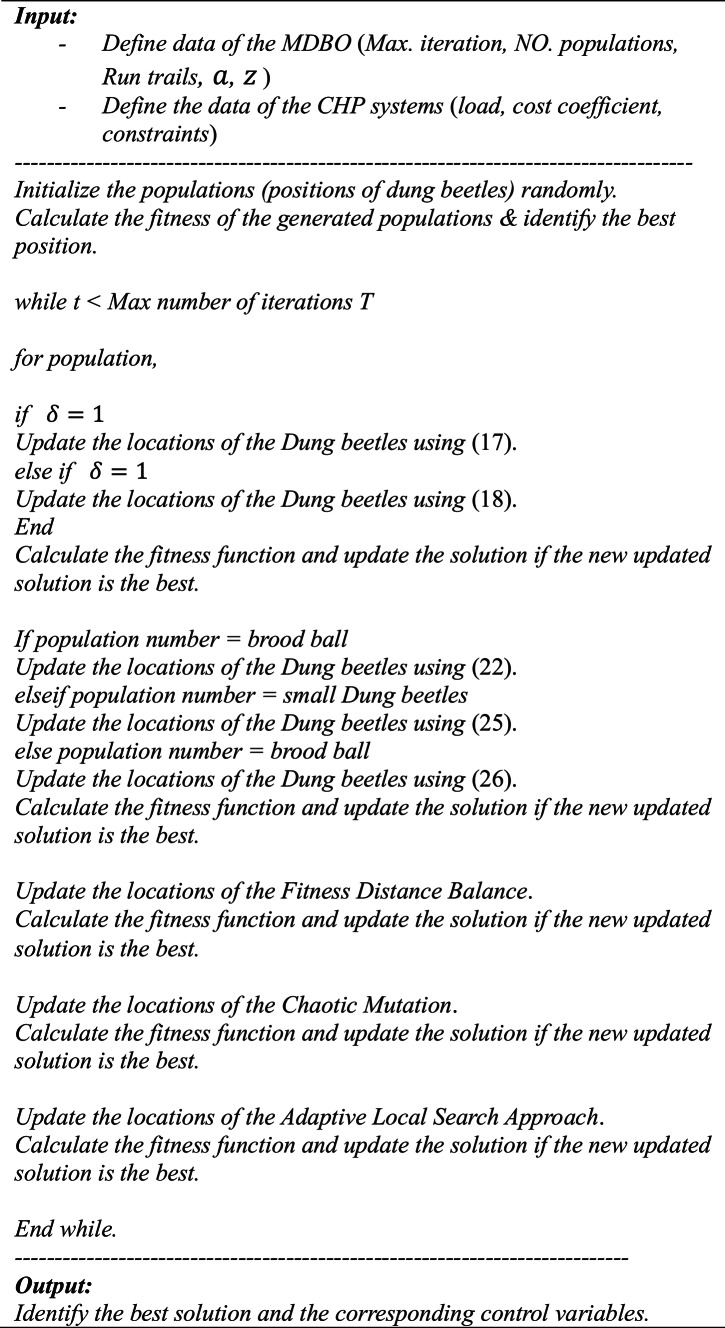
Pseudo code the proposed MDBO.

## Numerical results

### Validation and analysis of simulation

This section presents and evaluates the performance of the proposed MDBO in solving the CHPED problem for different test systems, including four, seven, twenty-four-unit, and forty-eight configurations. Initially, the MDBO is benchmarked against 23 standard test functions and the CEC-2019 test suite. The description of standard functions is given in ^60,61^ while the description of CEC-2019 functions is provided in ^62,63^. Its performance is compared with nine state-of-the-art optimization algorithms, namely: Whale Optimization Algorithm (WOA) ^64^, Sand Cat Swarm Optimization (SCSO) ^65^, Zebra Optimization Algorithm (ZOA)^66^, African Vultures Optimization Algorithm (AVOA) ^67^, Grey Wolf Optimizer (GWO) ^68^, Harris Hawks Optimization (HHO)^69^, Liver Cancer Algorithm (LCA) ^70^, and Dung Beetle Optimizer (DBO) ^41^. All simulations were executed using MATLAB 2021b on a Core i7 processor (2.5 GHz, 8 GB RAM). Table 1 summarizes the parameter settings of the comparative optimization algorithms.

**Table 1:** The selected parameters of the optimization methods.

${\boldsymbol{A}}{\boldsymbol{l}}{\boldsymbol{g}}{\boldsymbol{o}}{\boldsymbol{r}}{\boldsymbol{t}}{\boldsymbol{i}}{\boldsymbol{h}}{\boldsymbol{m}}{\boldsymbol{s}}$	Parameters
SCSO	${T}_{max}=300, Search Agent=30, Runs=25, rg=\left[\mathrm{2,0}\right], R=\left[- 2rg, 2rg\right]$
AVOA	${T}_{max}=300, Search Agent=30, Runs=25, probability parameters w=2.5,$ $L1 \& L2=0.8 \& 0.2; P1,P2 \& P3=0.6, 0.4 \& 0.6$
SCA	${T}_{max}=300, Search Agent=30, Runs=25, constant a=2, t=2$
HHO	${T}_{max}=300, Search Agent=30, Runs=25, {E}_{0}=[-\mathrm{1,1}], \beta =1.5$
GWO	${T}_{max}=300, Search Agent=30, Runs=25, a=[\mathrm{2,0}]$
LCA	${T}_{max}=300, Search Agent=30, Runs=25, p=0.03, \beta =3, w=0.8$
ZOA	${T}_{max}=300, Search Agent=30, Runs=25, a R=0.01$
WOA	${T}_{max}=300, Search Agent=30, Runs=25,$
DBO	${T}_{max}=300, Search Agent=30, Runs=25,$
MDBO	${T}_{max}=300, Search Agent=30, Runs=25, a=4, z=0.1$

### Benchmark validation

To validate the effectiveness of MDBO compared to the statistical outcomes of other methods (provided in Table 1), extensive experiments were conducted using two widely accepted benchmark suites. The statistical evaluation includes convergence curves, box plots, and non-parametric significance tests, such as the Wilcoxon rank-sum test and Friedman’s mean rank test.

#### Statistical analysis

The statistical performance of MDBO and the comparative algorithms is presented in Table 2 for the 23 standard benchmark functions and Table 3 for the CEC-2019 suite. The metrics considered include average, best, and worst, standard deviation and simulation time of the obtained results. Across most test functions, MDBO demonstrated superior performance in terms of average and best values, indicating its effectiveness and robustness. However, the computational time of the proposed MDBO is the highest compared to the original DBO and the other optimization algorithms due to the three integrated modifications.

**Table 2:** Statistical performance on 23 standard benchmark functions.

Function	Optimizer	Average	Best	Worst	SD	Time
F1	MDBO	0	0	4.3E-308	0	2.60
	DBO	9.72E-68	5.4E-101	2.43E-66	4.85E-67	0.65
	SCSO	1.14E-64	3.08E-77	2.85E-63	5.7E-64	7.71
	AVOA	8.1E-171	1.6E-226	2E-169	0	0.91
	SCA	263.4955	0.042398	2345.367	531.6366	0.57
	HHO	2.87E-59	1.1E-77	7.15E-58	1.43E-58	0.02
	GWO	4.5E-15	5.54E-16	3.37E-14	6.67E-15	0.61
	LLCA	0.399049	0.000723	5.780015	1.145176	0.02
	ZOA	1.8E-143	7E-155	4.4E-142	8.9E-143	0.41
	WOA	2.41E-41	1.17E-49	5.97E-40	1.19E-40	0.28
F2	MDBO	2.9E-180	0	4.7E-179	0	2.66
	DBO	6.84E-35	2.32E-49	9.63E-34	2.37E-34	0.68
	SCSO	2.58E-36	1E-39	4.89E-35	9.73E-36	7.69
	AVOA	4.72E-87	4E-117	9.38E-86	1.91E-86	0.89
	SCA	0.280991	0.045576	0.837105	0.214318	0.58
	HHO	1.51E-31	2.58E-38	3.54E-30	7.07E-31	0.02
	GWO	1.86E-09	4.69E-10	7.7E-09	1.55E-09	0.63
	LLCA	0.204908	0.02292	0.708115	0.169119	0.02
	ZOA	3.51E-77	4.92E-82	7.61E-76	1.52E-76	0.47
	WOA	1.41E-30	1.89E-34	1.08E-29	2.99E-30	0.30
F3	MDBO	0	0	0	0	6.00
	DBO	8.38E-28	2.2E-101	2.1E-26	4.19E-27	1.64
	SCSO	2.31E-58	8.35E-67	4.11E-57	8.35E-58	8.58
	AVOA	6E-124	1.4E-178	1.5E-122	3E-123	1.87
	SCA	14,357.94	3211.651	35,076.64	8015.715	1.60
	HHO	3.11E-44	8.95E-68	7.23E-43	1.45E-43	0.12
	GWO	0.121837	0.000284	0.908199	0.236811	1.57
	LLCA	44.05377	2.117984	215.7927	59.50837	0.09
	ZOA	1.33E-91	2.7E-108	2.32E-90	4.84E-91	2.32
	WOA	59,402.73	23,058.21	87,841.88	15,627.12	1.22
F4	MDBO	1.6E-120	0	1.6E-119	4.7E-120	2.63
	DBO	9.91E-30	2.77E-59	2.48E-28	4.95E-29	0.67
	SCSO	5.64E-30	5.37E-34	1.26E-28	2.51E-29	7.69
	AVOA	1.6E-82	4.2E-110	3.99E-81	7.98E-82	0.88
	SCA	43.14618	29.69865	60.21589	9.771157	0.56
	HHO	3.14E-30	4.71E-37	6.32E-29	1.28E-29	0.03
	GWO	0.000922	0.000169	0.004968	0.000999	0.62
	LLCA	0.088243	0.003476	0.345074	0.083944	0.02
	ZOA	1.99E-67	1.1E-71	2.37E-66	6.3E-67	0.42
	WOA	63.5515	0.553079	90.56709	25.58868	0.26
F5	MDBO	0.000976	3.02E-07	0.010354	0.002178	2.99
	DBO	26.47943	26.04447	27.69189	0.370186	0.78
	SCSO	28.0002	26.18425	28.85621	0.881745	7.77
	AVOA	0.000175	6.49E-06	0.000516	0.000158	1.05
	SCA	643,275.6	218.5034	5,424,867	1,389,638	0.72
	HHO	0.027941	3.65E-05	0.133945	0.03467	0.05
	GWO	27.31274	25.69477	28.76924	0.866912	0.73
	LLCA	2.730639	0.006208	13.22004	3.479955	0.03
	ZOA	28.60935	27.99514	28.8674	0.282854	0.67
	WOA	28.37393	27.71719	28.77789	0.301987	0.40
F6	MDBO	6.55E-05	5.43E-08	0.000383	0.000101	2.59
	DBO	0.059113	0.001317	0.526892	0.117914	0.62
	SCSO	2.09669	1.23559	3.477596	0.664548	7.61
	AVOA	1.32E-05	2.27E-06	6.76E-05	1.36E-05	0.88
	SCA	217.0573	7.860881	684.4872	226.8401	0.56
	HHO	0.000459	2.59E-07	0.004146	0.000864	0.03
	GWO	1.027095	0.251533	1.761527	0.384384	0.64
	LLCA	0.154203	0.006161	0.829026	0.232054	0.02
	ZOA	3.043646	1.090045	4.42943	0.755135	0.43
	WOA	0.728101	0.256194	1.364056	0.294	0.27
F7	MDBO	0.000152	1.22E-05	0.000638	0.000139	3.21
	DBO	0.00164	7.44E-05	0.005196	0.00132	1.32
	SCSO	0.000572	1.56E-06	0.004074	0.00091	8.27
	AVOA	0.000349	3.41E-05	0.001726	0.000423	1.55
	SCA	0.359998	0.029193	1.873522	0.415875	1.24
	HHO	0.000217	1.08E-05	0.000574	0.000168	0.09
	GWO	0.003896	0.001256	0.007079	0.001628	1.34
	LLCA	0.000798	2.09E-05	0.002605	0.000732	0.07
	ZOA	0.000156	1.41E-05	0.000531	0.000117	1.73
	WOA	0.01072	7.27E-05	0.047334	0.012754	0.95
F8	MDBO	-12,569.5	-12,569.5	-12,569.5	7.18E-06	3.04
	DBO	-7740.9	-11,521.4	-6014.42	1422.855	0.87
	SCSO	-6717.16	-8012.83	-5145.53	684.4666	7.84
	AVOA	-12,400.8	-12,569.5	-11,300.2	320.4961	1.09
	SCA	-3687.92	-4538.72	-3295.75	341.7	0.74
	HHO	-12,550.2	-12,569.5	-12,132.2	87.09876	0.05
	GWO	-5723.73	-8049.65	-3562.57	1011.549	0.77
	LLCA	-8024.79	-12,569.4	-2095.38	4310.2	0.03
	ZOA	-6164.05	-7043.19	-5159.07	545.0773	0.70
	WOA	-9609.52	-12,569.5	-6084.65	1860.547	0.39
F9	MDBO	0	0	0	0	2.44
	DBO	0	0	0	0	0.70
	SCSO	0	0	0	0	7.67
	AVOA	0	0	0	0	0.90
	SCA	81.18127	2.11015	166.5803	42.71725	0.67
	HHO	0	0	0	0	0.04
	GWO	7.543239	3.64E-12	19.08102	5.410151	0.68
	LLCA	16.44385	0.000843	251.8957	58.2602	0.03
	ZOA	0	0	0	0	0.50
	WOA	2.27E-15	0	5.68E-14	1.14E-14	0.31
F10	MDBO	4.44E-16	4.44E-16	4.44E-16	0	2.68
	DBO	4.44E-16	4.44E-16	4.44E-16	0	0.72
	SCSO	4.44E-16	4.44E-16	4.44E-16	0	7.68
	AVOA	4.44E-16	4.44E-16	4.44E-16	0	0.90
	SCA	14.78978	0.052012	20.4038	8.173789	0.72
	HHO	4.44E-16	4.44E-16	4.44E-16	0	0.04
	GWO	1.26E-08	4.57E-09	2.59E-08	6.15E-09	0.68
	LLCA	0.148961	0.011435	0.372979	0.111602	0.03
	ZOA	4.44E-16	4.44E-16	4.44E-16	0	0.47
	WOA	4.28E-15	4.44E-16	7.55E-15	2.03E-15	0.32
F11	MDBO	0	0	0	0	2.84
	DBO	0	0	0	0	0.87
	SCSO	0	0	0	0	7.77
	AVOA	0	0	0	0	1.03
	SCA	2.492506	0.659017	6.973651	1.877228	0.82
	HHO	0	0	0	0	0.05
	GWO	0.004378	2.78E-15	0.040893	0.010977	0.78
	LLCA	0.39276	0.025455	1.022877	0.371915	0.03
	ZOA	0	0	0	0	0.66
	WOA	8.88E-18	0	1.11E-16	3.07E-17	0.42
F12	MDBO	9.1E-07	2.66E-08	4.78E-06	1.34E-06	8.89
	DBO	0.001346	3.5E-05	0.0097	0.002525	2.53
	SCSO	0.12207	0.054102	0.2254	0.053023	9.44
	AVOA	6.33E-07	6.55E-08	4.21E-06	9.45E-07	2.75
	SCA	958,453.7	1.825575	14,146,578	2,894,310	2.42
	HHO	1.55E-05	2.59E-08	6.3E-05	1.82E-05	0.20
	GWO	0.059873	0.022789	0.206004	0.04305	2.42
	LLCA	0.001377	2.34E-05	0.007325	0.00178	0.16
	ZOA	0.2120v 66	0.103886	0.406169	0.075033	4.05
	WOA	0.100678	0.012778	1.493783	0.290832	2.07
F13	MDBO	4.98E-07	4.49E-10	3.14E-06	6.95E-07	8.88
	DBO	1.124984	0.204333	2.324522	0.581429	2.53
	SCSO	2.380166	1.492835	2.888264	0.406588	9.53
	AVOA	0.000442	1.24E-08	0.011038	0.002207	2.68
	SCA	1,554,695	44.74112	15,354,981	3,203,404	2.43
	HHO	0.000162	6.9E-08	0.000907	0.000251	0.21
	GWO	0.748238	0.314032	1.361331	0.251498	2.51
	LLCA	0.013338	0.000183	0.068833	0.015344	0.16
	ZOA	2.339419	1.616163	2.880444	0.25603	3.92
	WOA	0.699558	0.283219	1.320056	0.286263	2.06
F14	MDBO	0.998004	0.998004	0.998004	4.53E-17	11.59
	DBO	1.39589	0.998004	5.928845	1.103398	3.35
	SCSO	3.479096	0.998004	10.76318	2.887133	3.48
	AVOA	1.864953	0.998004	10.76318	2.023076	3.28
	SCA	2.120402	0.998005	2.982105	0.993044	3.16
	HHO	1.941964	0.998004	10.76318	2.305983	0.30
	GWO	5.660315	0.998004	12.67051	4.180229	2.94
	LLCA	12.77705	0.998004	58.96676	15.25463	0.23
	ZOA	3.384844	0.998004	6.903336	2.29754	5.79
	WOA	3.085985	0.998004	10.76318	2.741896	3.03
F15	MDBO	0.000307	0.000307	0.000308	2.17E-08	2.11
	DBO	0.000902	0.000307	0.001489	0.000386	0.58
	SCSO	0.000544	0.000307	0.001506	0.000323	1.24
	AVOA	0.000472	0.000308	0.000767	0.00017	0.59
	SCA	0.001165	0.000554	0.001593	0.000385	0.26
	HHO	0.000424	0.000308	0.001514	0.000245	0.03
	GWO	0.005372	0.000364	0.020363	0.0086	0.26
	LLCA	0.001729	0.000376	0.008506	0.001499	0.02
	ZOA	0.002012	0.000308	0.020363	0.005528	0.36
	WOA	0.001345	0.000315	0.01928	0.003753	0.24
F16	MDBO	-1.03163	-1.03163	-1.03163	6.25E-16	2.22
	DBO	-1.03163	-1.03163	-1.03163	5.46E-16	0.58
	SCSO	-1.03163	-1.03163	-1.03163	1.94E-09	0.76
	AVOA	-1.03163	-1.03163	-1.03163	1.23E-14	0.61
	SCA	-1.03153	-1.03163	-1.03111	0.000127	0.27
	HHO	-1.03163	-1.03163	-1.03163	2.66E-08	0.03
	GWO	-1.03163	-1.03163	-1.03163	6.54E-08	0.26
	LLCA	-0.81166	-1.01816	-0.36389	0.172334	0.02
	ZOA	-1.03163	-1.03163	-1.03163	3.04E-10	0.39
	WOA	-1.03163	-1.03163	-1.03163	9.34E-09	0.24
F17	MDBO	0.397887	0.397887	0.397887	0	1.74
	DBO	0.397887	0.397887	0.397887	0	0.50
	SCSO	0.397887	0.397887	0.397888	9.3E-08	0.69
	AVOA	0.397887	0.397887	0.397887	3.56E-16	0.51
	SCA	0.400459	0.397889	0.406473	0.002222	0.19
	HHO	0.397897	0.397887	0.397998	2.23E-05	0.02
	GWO	0.39789	0.397887	0.397898	2.42E-06	0.21
	LLCA	0.554313	0.401418	0.846658	0.126877	0.01
	ZOA	0.397887	0.397887	0.397888	8.37E-08	0.28
	WOA	0.397956	0.397887	0.398793	0.000187	0.18
F18	MDBO	3	3	3	1.37E-15	1.87
	DBO	3.000006	3	3.000155	3.11E-05	0.51
	SCSO	3.000018	3	3.000053	1.38E-05	0.69
	AVOA	3.000012	3	3.000184	3.89E-05	0.52
	SCA	3.000147	3.000001	3.000639	0.000199	0.19
	HHO	3.000001	3	3.000005	1.16E-06	0.02
	GWO	3.000117	3.000002	3.000624	0.000128	0.19
	LLCA	21.68911	3.008434	33.0415	10.72511	0.02
	ZOA	3.000004	3	3.000029	8.13E-06	0.29
	WOA	3.000231	3	3.002287	0.000518	0.19
F19	MDBO	-3.86278	-3.86278	-3.86278	2.25E-15	2.27
	DBO	-3.86137	-3.86278	-3.8549	0.002978	0.65
	SCSO	-3.86063	-3.86278	-3.8549	0.003393	1.04
	AVOA	-3.86278	-3.86278	-3.86278	5.25E-10	0.61
	SCA	-3.85382	-3.86195	-3.84438	0.004804	0.28
	HHO	-3.85974	-3.86278	-3.85236	0.003042	0.03
	GWO	-3.86119	-3.86278	-3.8549	0.002646	0.29
	LLCA	-3.36827	-3.80288	-2.45345	0.267466	0.02
	ZOA	-3.83081	-3.86278	-3.08975	0.154393	0.46
	WOA	-3.85701	-3.86278	-3.83297	0.008251	0.28
F20	MDBO	-3.26017	-3.322	-3.2031	0.060624	2.29
	DBO	-3.23481	-3.322	-2.84042	0.115604	0.61
	SCSO	-3.22281	-3.32199	-3.08356	0.077579	1.81
	AVOA	-3.27714	-3.322	-3.17133	0.061328	0.65
	SCA	-2.83682	-3.1189	-1.90746	0.371652	0.31
	HHO	-3.05388	-3.20815	-2.70685	0.134233	0.03
	GWO	-3.28422	-3.32199	-3.14104	0.062807	0.33
	LLCA	-1.63323	-2.58243	-0.602	0.500415	0.02
	ZOA	-3.29413	-3.32199	-3.1668	0.056042	0.42
	WOA	-3.19568	-3.32176	-2.49868	0.20542	0.27
F21	MDBO	-10.1532	-10.1532	-10.1532	4.05E-15	2.37
	DBO	-7.71547	-10.1532	-2.63047	2.86826	0.65
	SCSO	-5.1039	-10.153	-0.88199	1.838164	1.33
	AVOA	-10.1532	-10.1532	-10.1532	6.46E-11	0.64
	SCA	-2.8884	-5.92159	-0.49652	1.95182	0.31
	HHO	-5.43515	-9.92996	-5.02399	1.344436	0.03
	GWO	-7.84121	-10.1515	-2.62984	3.22824	0.32
	LLCA	-5.0508	-5.05516	-5.04041	0.004232	0.02
	ZOA	-9.94783	-10.1532	-5.0552	1.019318	0.49
	WOA	-8.11224	-10.1503	-2.61876	2.601389	0.30
F22	MDBO	-10.4029	-10.4029	-10.4029	3.46E-15	2.45
	DBO	-7.49831	-10.4029	-2.7659	3.165465	0.69
	SCSO	-6.31039	-10.4029	-3.72429	2.362753	1.36
	AVOA	-10.4029	-10.4029	-10.4029	3.19E-11	0.74
	SCA	-2.8777	-7.35255	-0.52094	1.83724	0.37
	HHO	-4.98394	-5.08762	-2.71983	0.471768	0.04
	GWO	-9.76215	-10.4016	-5.08755	1.757029	0.36
	LLCA	-4.43919	-5.76984	-0.70081	1.639422	0.02
	ZOA	-8.91293	-10.4029	-5.08766	2.43183	0.54
	WOA	-6.54641	-10.4004	-1.83413	2.769731	0.33
F23	MDBO	-10.5364	-10.5364	-10.5364	2.83E-15	2.68
	DBO	-7.12344	-10.5364	-1.85948	3.227422	0.76
	SCSO	-7.17498	-10.5364	-1.67654	3.170916	1.41
	AVOA	-10.5364	-10.5364	-10.5364	1.45E-10	0.73
	SCA	-3.36804	-7.28756	-0.94372	1.734708	0.41
	HHO	-5.43194	-10.5013	-2.78705	1.52799	0.04
	GWO	-9.99176	-10.5359	-2.42155	1.911391	0.43
	LLCA	-4.66501	-5.12847	-1.10827	1.270957	0.03
	ZOA	-9.45462	-10.5364	-5.12811	2.207726	0.66
	WOA	-5.25408	-10.464	-1.65188	2.783755	0.39

**Table 3:** Statistical performance on CEC-2019 benchmark suite.

Function	Optimizer	Average	Best	Worst	SD
CEC01	MDBO	39,016.14	34,411.75	43,169.87	2105.715
	DBO	7.62E + 09	40,395.89	9.06E + 10	1.88E + 10
	SCSO	45,303.2	37,342.95	52,101.24	3349.062
	AVOA	48,128.44	39,883.45	66,869.17	6624.212
	SCA	1.5E + 10	17,816,487	5.46E + 10	1.54E + 10
	HHO	53,070.93	42,814.57	71,942.99	6415.007
	GWO	3.25E + 08	378,395.9	2.43E + 09	5.09E + 08
	LLCA	4.76E + 08	2,901,991	3.04E + 09	6.76E + 08
	ZOA	53,922.17	38,822.37	303,936.9	52,160.12
	WOA	4.68E + 10	917,472.1	2.34E + 11	6.14E + 10
CEC02	MDBO	18.34286	18.34286	18.34286	6.8E-15
	DBO	18.34286	18.34286	18.34286	7.36E-15
	SCSO	18.38356	18.34299	18.69579	0.111622
	AVOA	18.35014	18.34286	18.52503	0.036434
	SCA	18.54482	18.43479	18.73487	0.105685
	HHO	18.36684	18.34528	18.39327	0.01001
	GWO	18.34459	18.34357	18.34541	0.000448
	LLCA	54.86242	21.50352	231.8426	48.38501
	ZOA	18.49826	18.34299	18.71111	0.126184
	WOA	18.40095	18.34502	19.25568	0.18299
CEC03	MDBO	13.7024	13.7024	13.7024	1.17E-10
	DBO	13.70241	13.7024	13.70246	1.47E-05
	SCSO	13.70241	13.7024	13.70245	8.7E-06
	AVOA	13.7024	13.7024	13.7024	1.25E-09
	SCA	13.7026	13.70241	13.70287	0.000138
	HHO	13.70242	13.7024	13.70246	1.38E-05
	GWO	13.7024	13.7024	13.70241	2.07E-06
	LLCA	13.70615	13.70309	13.70828	0.0012
	ZOA	13.70242	13.7024	13.70249	2.6E-05
	WOA	13.70241	13.7024	13.70241	1.6E-06
CEC04	MDBO	29.85474	8.959667	97.51024	19.83701
	DBO	220.7204	14.92957	1015.624	244.1118
	SCSO	613.7674	50.18866	3636.579	1034.114
	AVOA	149.2044	42.78872	485.6124	88.69863
	SCA	1851.652	451.6714	4150.808	905.1663
	HHO	535.2613	105.1879	1540.745	387.6402
	GWO	266.9631	38.01213	2500.612	575.3096
	LLCA	20,330.79	7853.9	37,155.17	7799.409
	ZOA	2305.747	163.7477	6739.558	2150.801
	WOA	645.6983	191.5316	1604.924	341.9346
CEC05	MDBO	2.124728	2.01478	2.406392	0.086375
	DBO	2.492938	2.063961	3.186548	0.372018
	SCSO	2.405775	2.130367	2.970051	0.181064
	AVOA	2.440592	2.021441	3.197585	0.336844
	SCA	3.310139	3.119241	3.901402	0.148492
	HHO	3.632302	2.610448	6.383734	0.961818
	GWO	2.328122	2.05071	2.775489	0.212661
	LLCA	6.696336	4.06465	8.660347	1.268306
	ZOA	2.93425	2.230416	4.813291	0.586996
	WOA	2.996838	2.346267	3.902953	0.418643
CEC06	MDBO	11.58759	10.46498	12.75928	0.618497
	DBO	11.64044	8.735864	14.00364	1.408789
	SCSO	9.363111	5.147488	11.78259	1.309391
	AVOA	7.284897	4.121767	9.959604	1.67785
	SCA	12.45368	11.10663	13.59683	0.599712
	HHO	10.45998	8.319398	12.09209	1.110967
	GWO	12.48719	11.00667	13.47829	0.546948
	LLCA	13.84206	11.10887	14.78218	0.850922
	ZOA	9.50984	6.753023	11.04023	1.014711
	WOA	10.74807	8.324786	12.54784	1.094149
CEC07	MDBO	321.2115	-50.4945	794.171	254.9438
	DBO	487.4334	27.31206	1080.865	301.7011
	SCSO	421.11	119.9385	1045.866	224.3932
	AVOA	496.9816	189.1112	873.288	202.3161
	SCA	735.2225	288.1046	1089.998	184.2583
	HHO	388.3867	-0.78057	855.9468	215.9372
	GWO	453.6227	-4.89515	1168.588	318.2756
	LLCA	1571.23	736.5539	2095.032	322.3756
	ZOA	137.5846	-51.2802	322.0822	95.49307
	WOA	636.5847	207.4383	1158.098	231.9127
CEC08	MDBO	5.261581	3.748251	6.407944	0.766368
	DBO	5.873945	4.000587	6.870756	0.740729
	SCSO	5.415661	3.939619	6.818807	0.723617
	AVOA	5.650311	3.94172	6.464638	0.652519
	SCA	6.253344	4.97736	6.704291	0.381285
	HHO	6.041456	5.527006	6.875936	0.338751
	GWO	5.328957	3.083785	7.044592	1.179707
	LLCA	7.444995	6.742793	8.038485	0.300896
	ZOA	4.964551	3.643649	5.699074	0.53681
	WOA	5.884133	3.793143	7.13807	0.72387
CEC09	MDBO	3.442045	3.359692	3.730953	0.078477
	DBO	3.925061	3.481047	4.96801	0.447547
	SCSO	31.33546	3.966517	353.06	86.53066
	AVOA	4.730627	3.427058	6.890969	0.797481
	SCA	170.9786	16.29902	500.482	123.3493
	HHO	4.98935	4.025844	6.02036	0.596838
	GWO	5.611083	4.251489	7.412887	0.786178
	LLCA	2991.13	1279.295	4352.486	858.8772
	ZOA	76.1216	5.167582	374.5694	129.3391
	WOA	7.432919	3.810137	15.36802	3.2525
CEC10	MDBO	21.4116	21.20165	21.58469	0.088458
	DBO	21.51084	21.20485	21.68339	0.127139
	SCSO	21.22132	20.98527	21.44041	0.131378
	AVOA	21.06712	20.99499	21.2978	0.089158
	SCA	21.51281	21.32702	21.6979	0.10893
	HHO	21.33995	21.12383	21.56835	0.140039
	GWO	20.6282	7.284401	21.64525	3.244834
	LLCA	21.77397	21.49397	21.93042	0.108356
	ZOA	20.70921	9.711443	21.33444	2.301684
	WOA	21.32411	21.10008	21.55974	0.13122

#### Convergence analysis

**Fig. 2:**
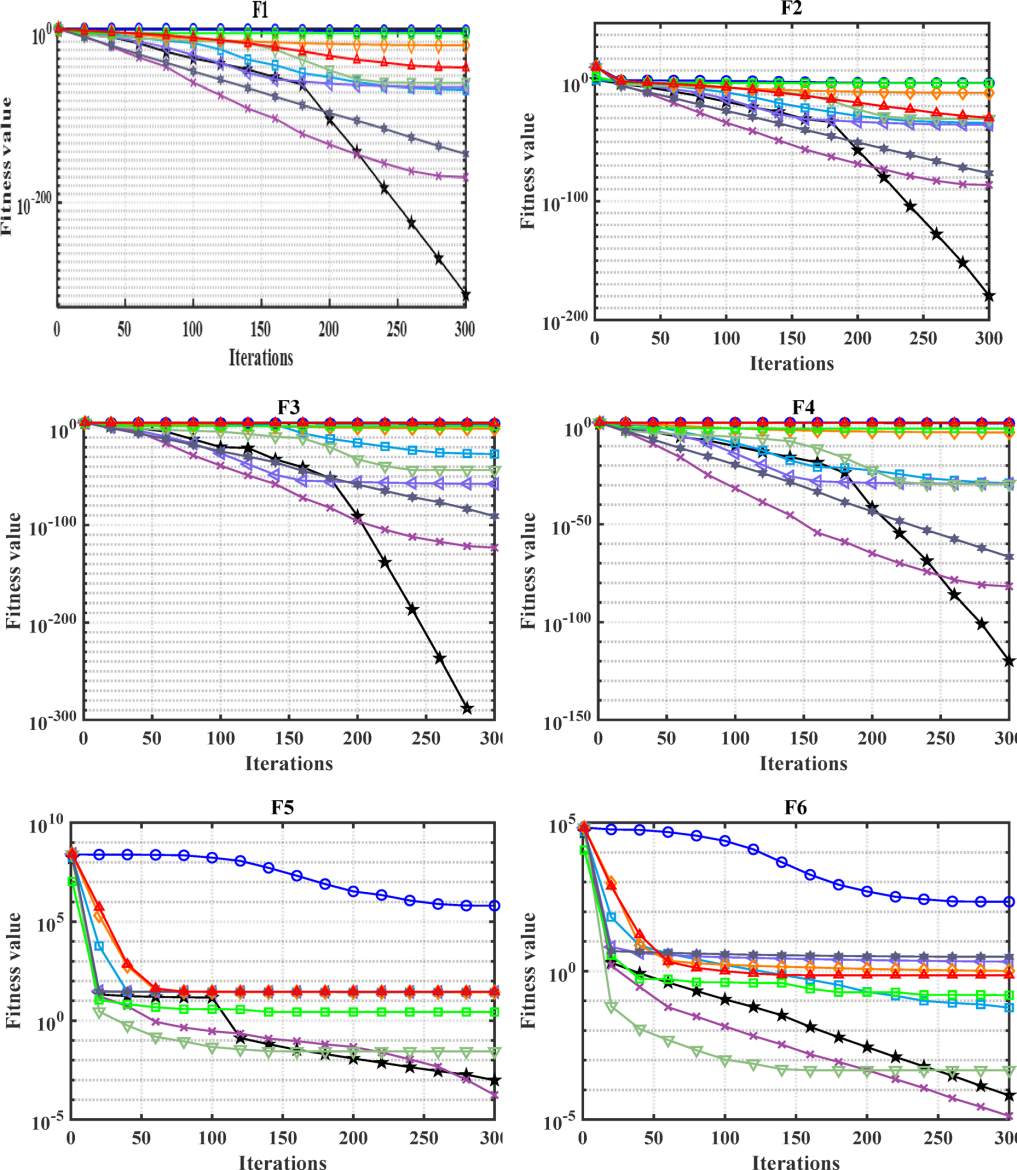

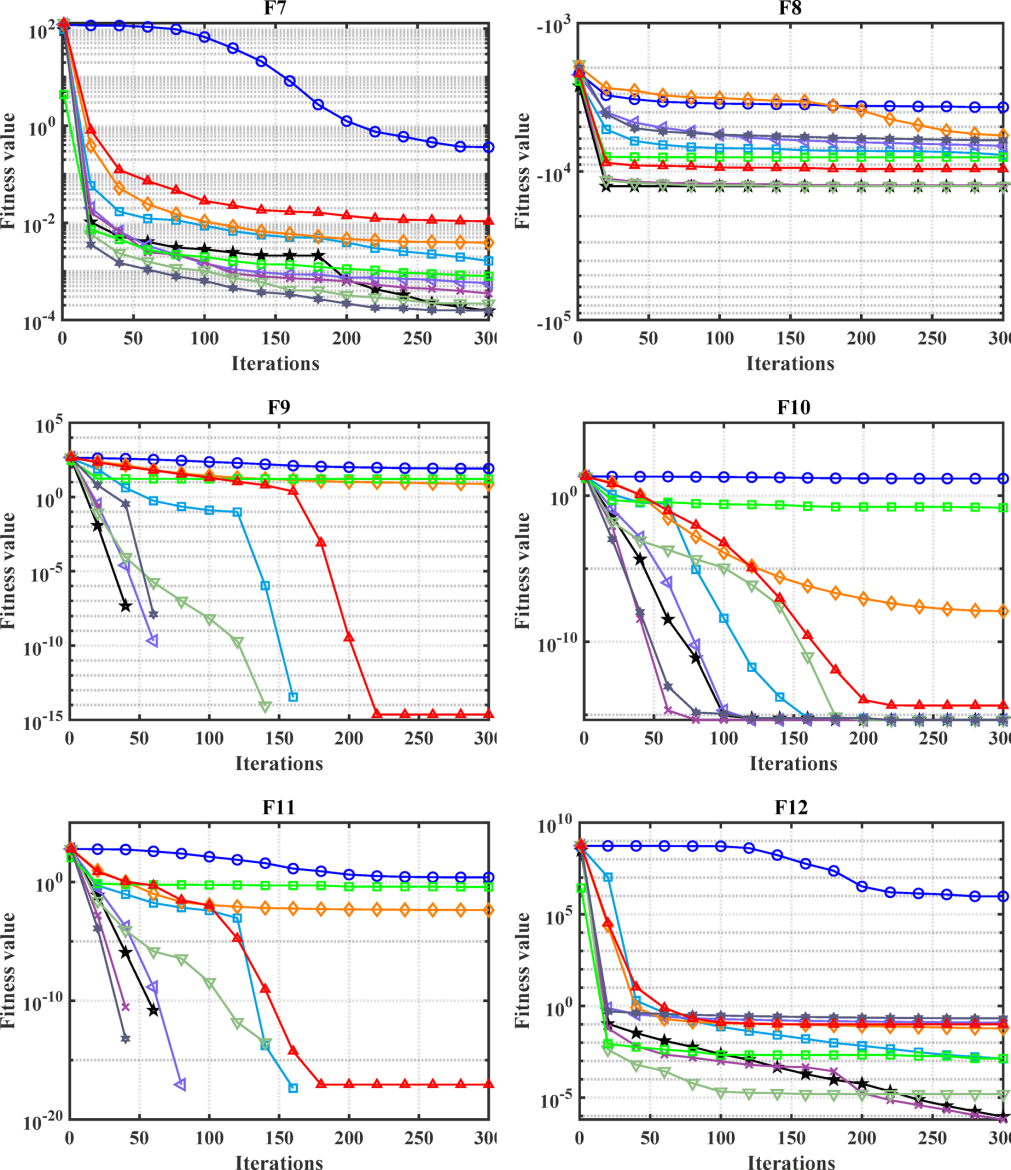

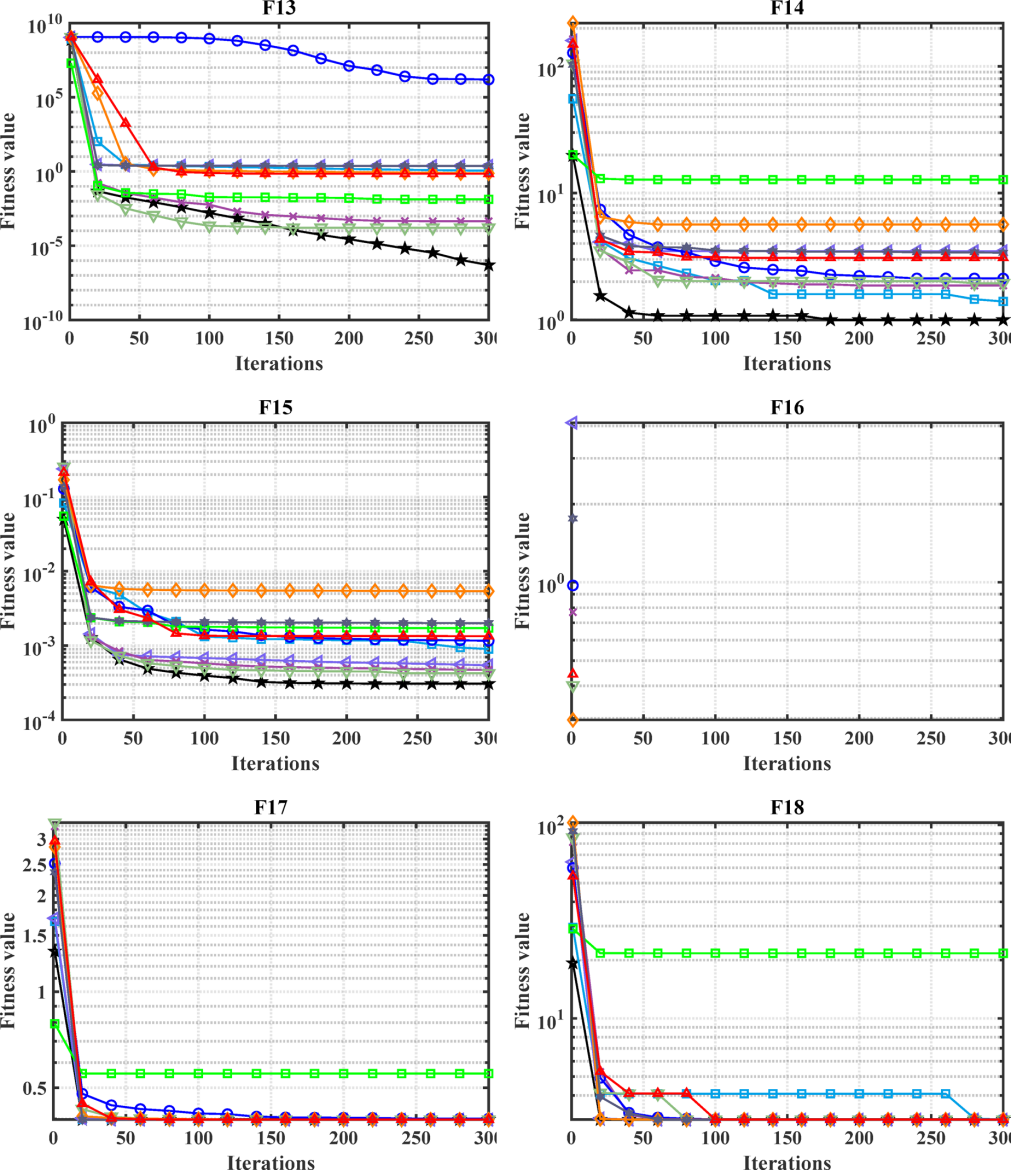

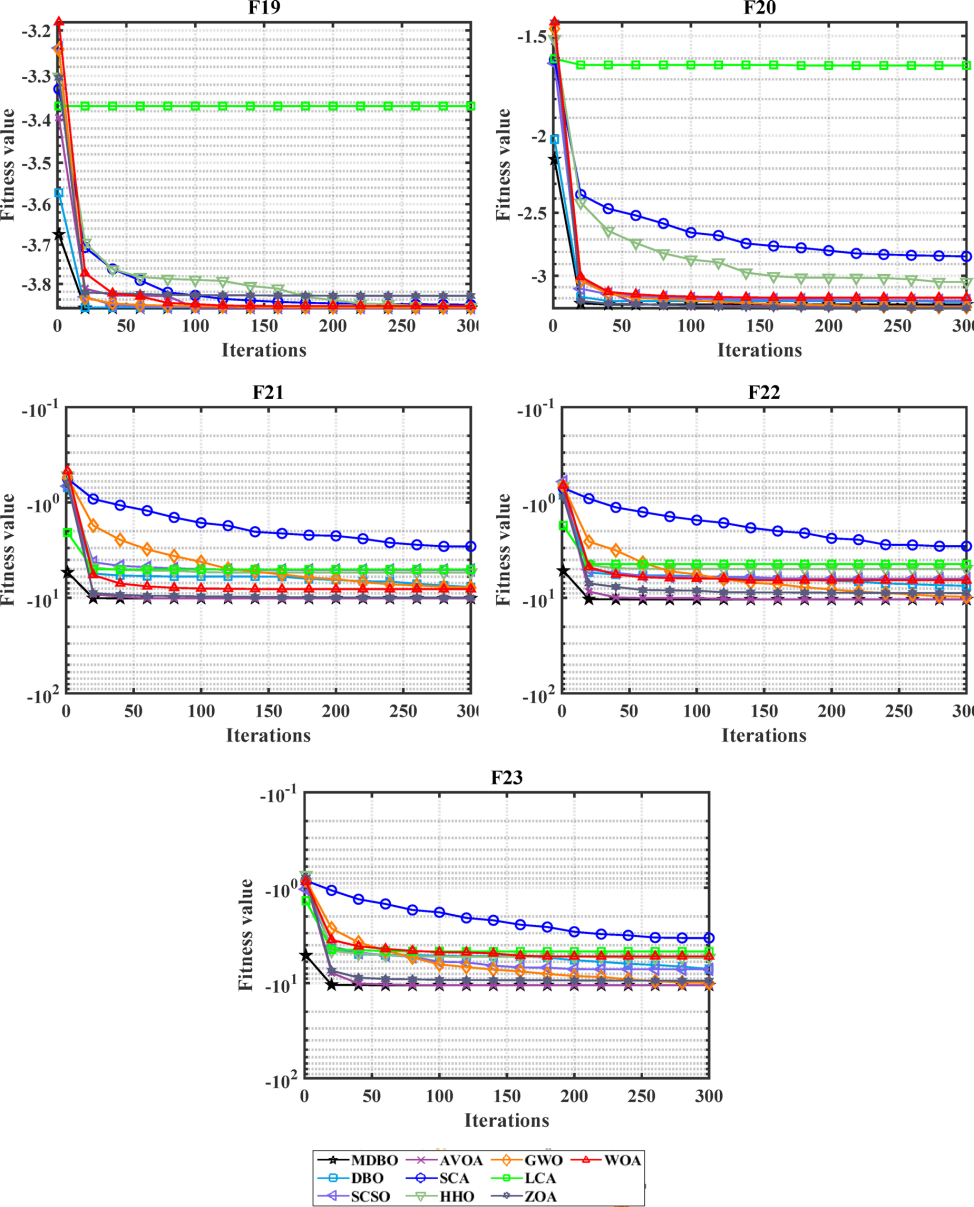
Convergence response of the proposed and other state-of-the-art techniques using standard 54 benchmark functions.

**Fig. 3:**
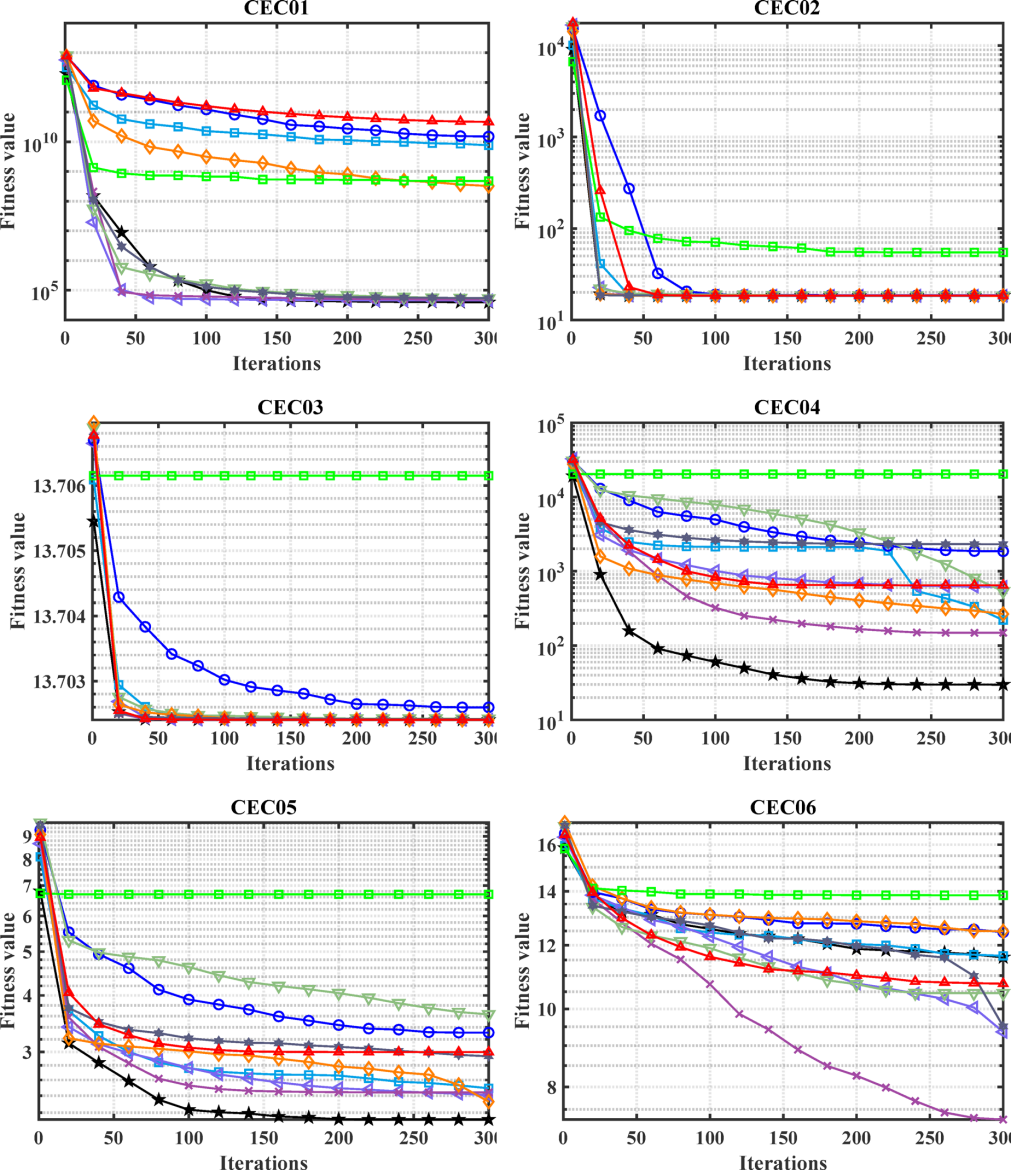

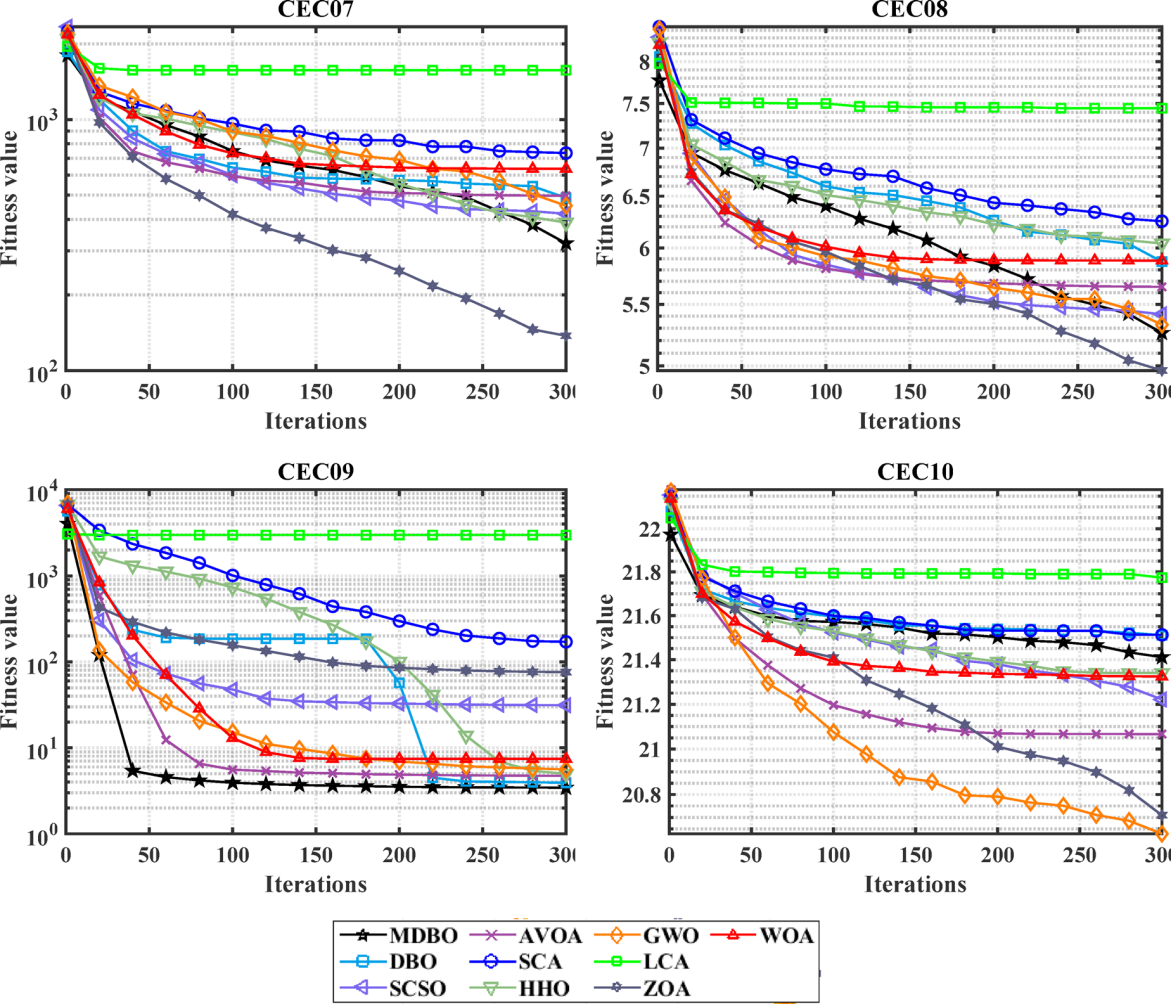
Convergence response of the proposed and other state-of-the-art techniques using CEC-2019 test-suit.

The convergence carve of the comparative optimizers for the standard benchmark functions and CEC-2019 are displayed in Figs. 2 and 3, respectively. According to Fig. 2, the proposed MDBO has stable and fast convergence characteristics compared to the other optimization algorithms for most benchmark functions where there is notable difference especially for F1 to F6, F14, F15, F21, F22, and F23 while the convergence of F5, F6, and F12 the AVOA is the best and for F9, the WOA is the best. Likewise, the convergence of the MDBO is the best compared to other optimization techniques for CEC-2019 functions as depicted in Fig. 3, except the AVOA is the best for CEC06 and ZOA is the best for CEC08, and CEC09 while the GWO is the best for CEC10.

#### Boxplot analysis

**Fig. 4:**
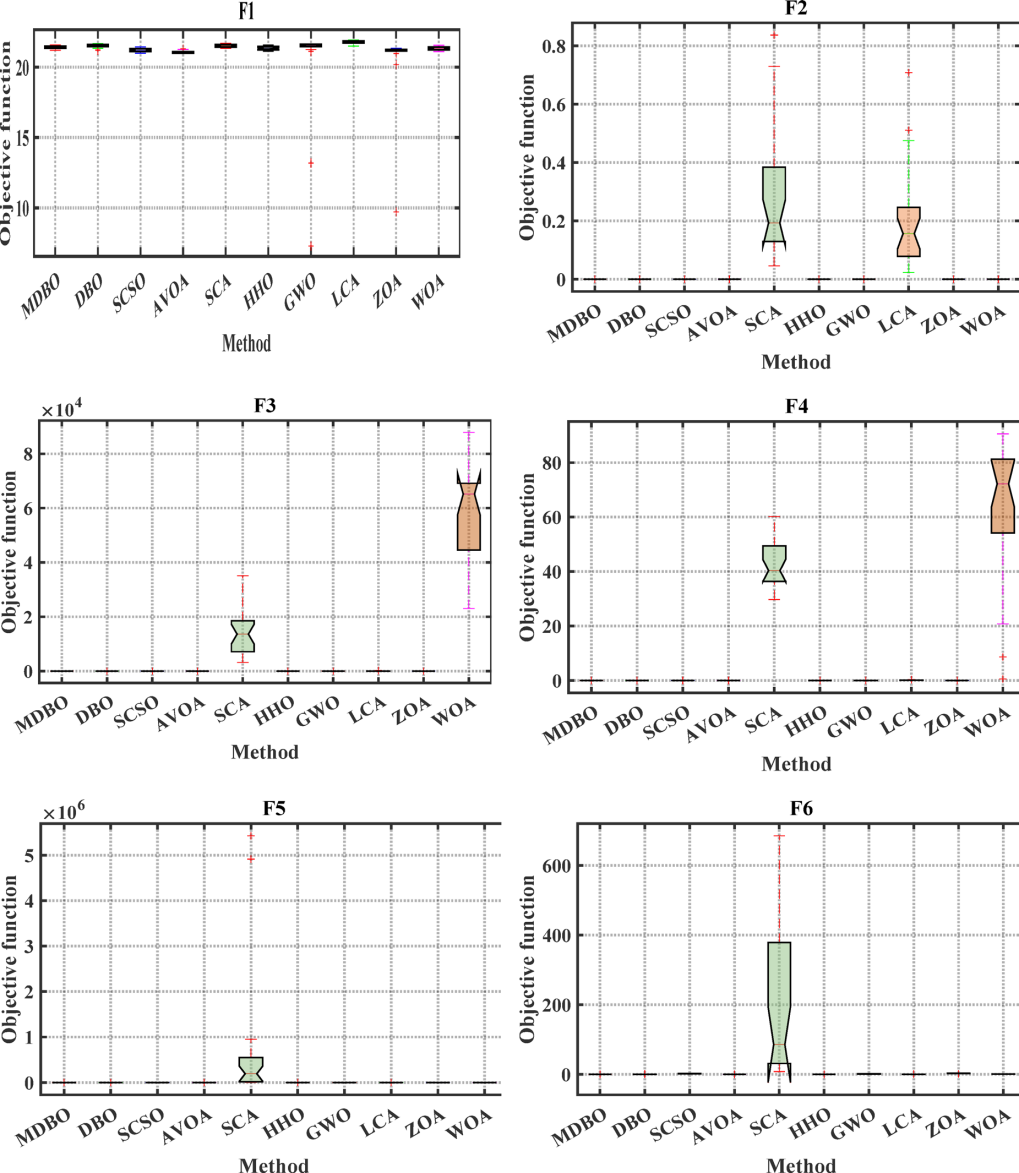

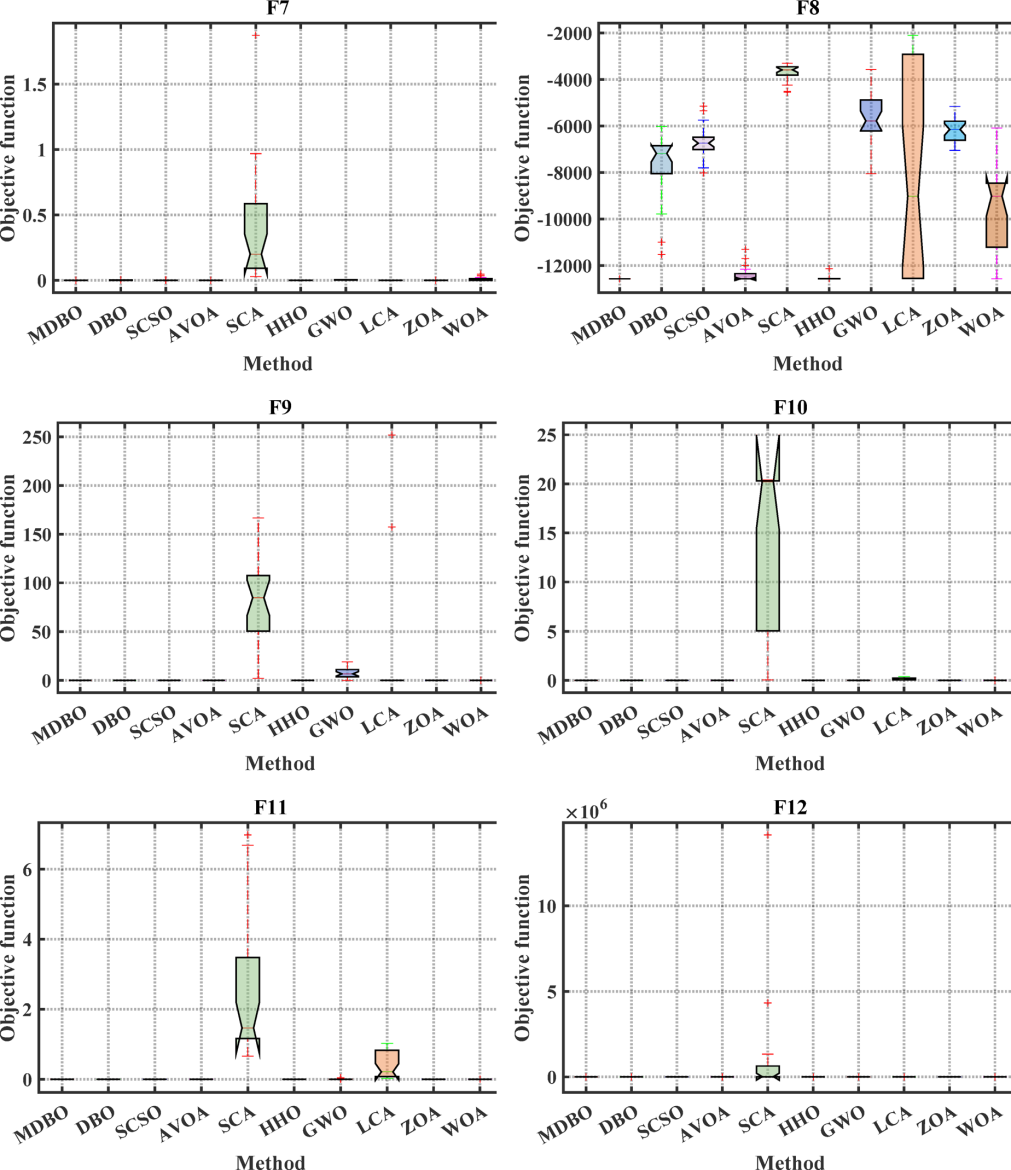

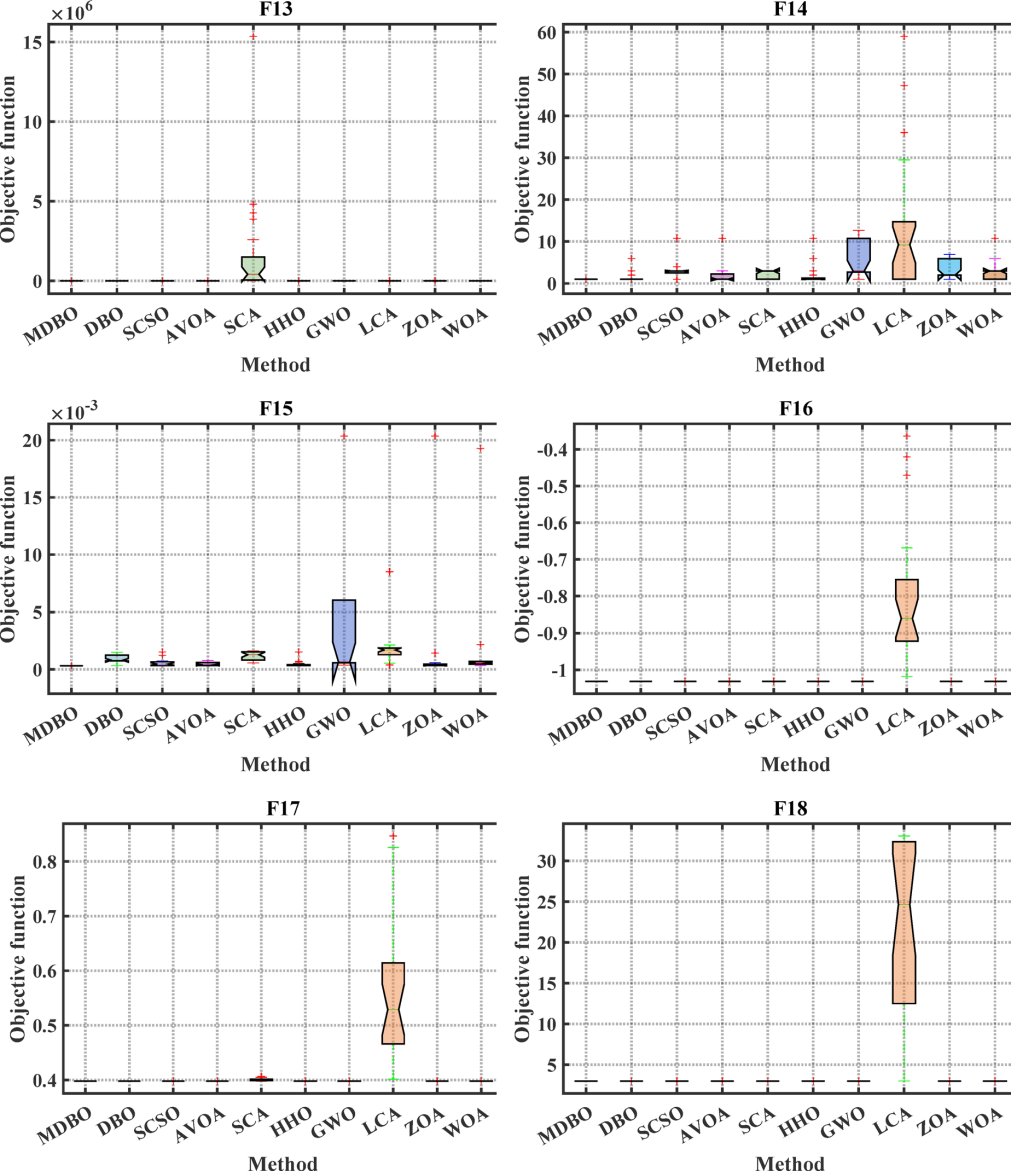

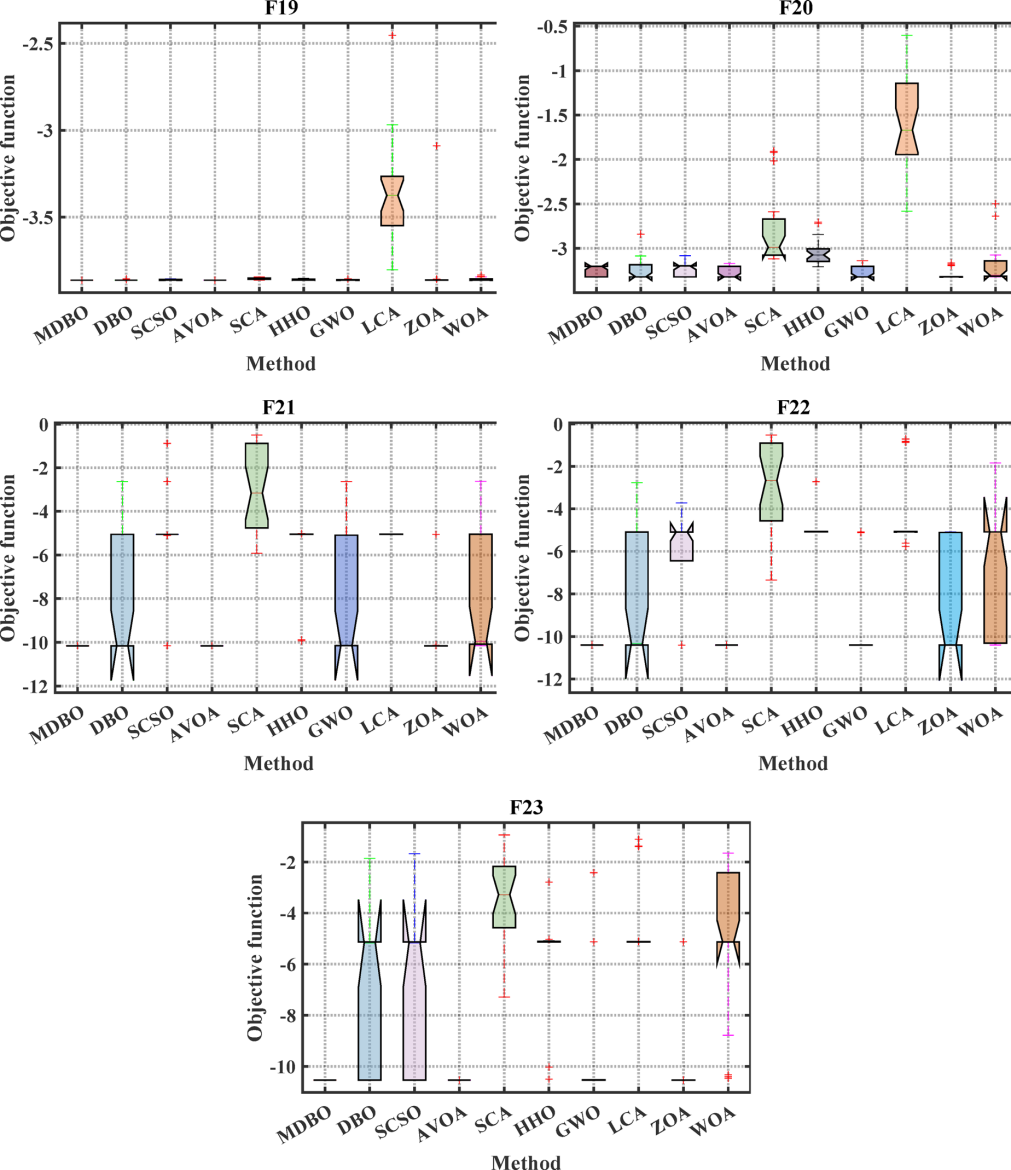
Boxplot response of the proposed and other state-of-the-art techniques using standard benchmark functions.

**Fig. 5:**
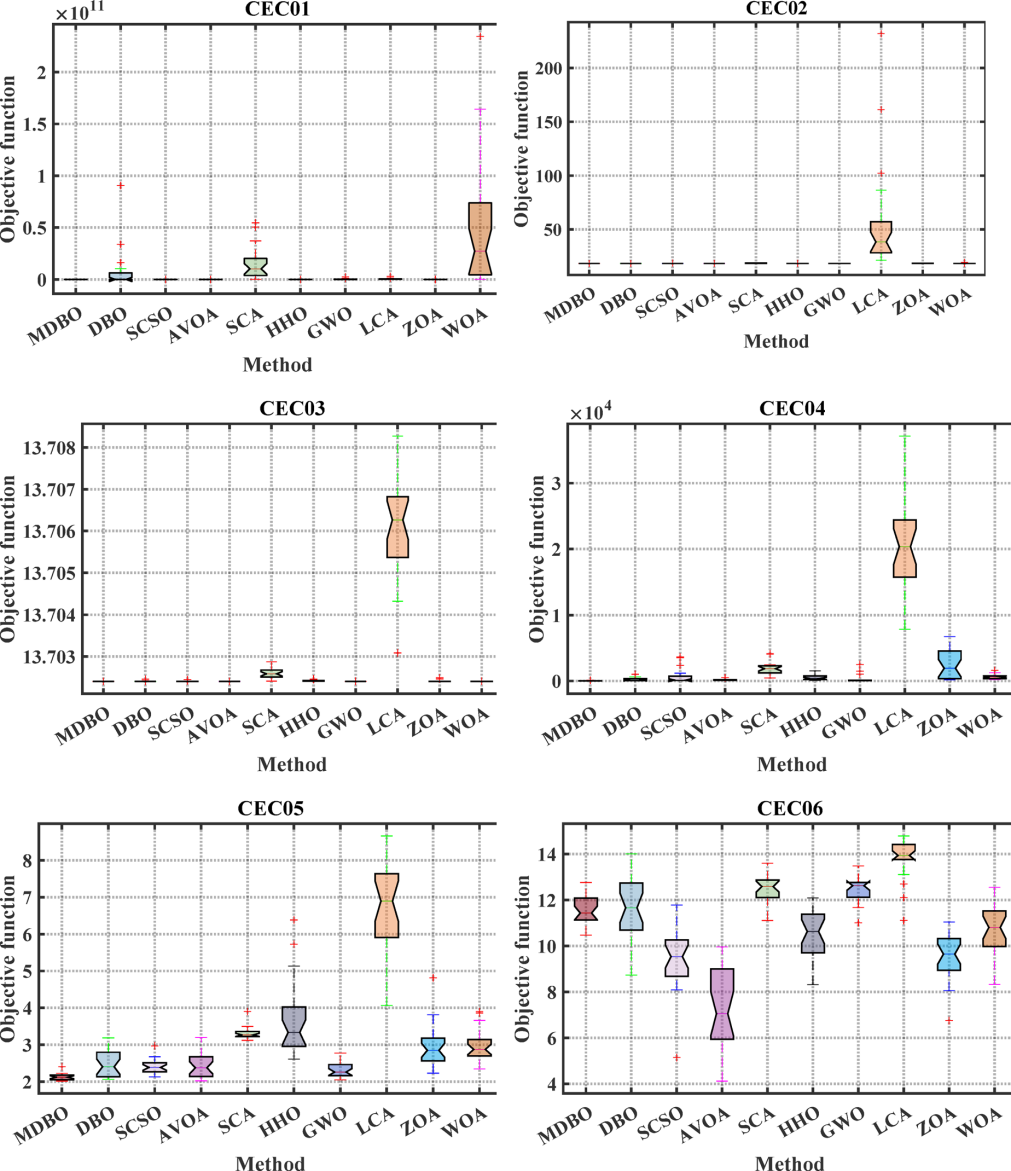

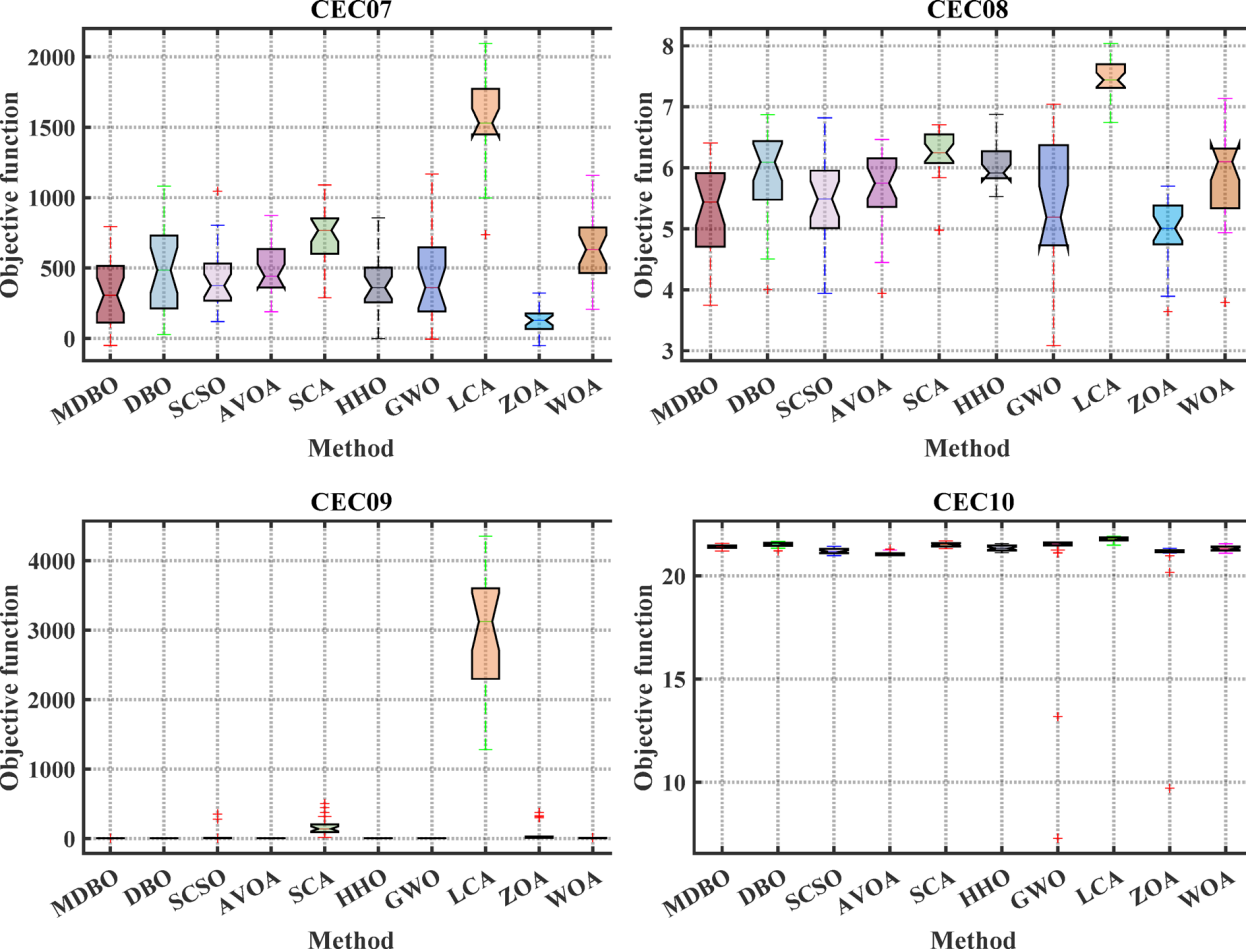
Boxplot response of the proposed and other state-of-the-art techniques using CEC-2019 test-suit.

Boxplot is a visual representation of obtained results by the comparative optimizers to test the performance and verify the effectiveness of the proposed optimizer. It is worth mentioning here that the narrowest boxplot means that there is no massive difference between the results obtained. Figure 4 and Fig. 5 shows the boxplot of the comparative optimizers for the classic and CEC-2019 functions, respectively. As depicted in Figs. 4 and 5, the MDBO has the narrowest boxplot for most functions.

#### Wilcoxon rank-sum test

The Wilcoxon rank-sum test, also known as the Mann–Whitney U test, is applied to statistically verify significant differences in performance. This non-parametric test is suitable for small or non-normally distributed datasets, comparing medians instead of means. The *p*-values of Wilcoxon test results which obtained from testing both benchmark suites are summarized in Table 4 for standard benchmarks and Table 5 for CEC-2019 suite. Most comparisons show statistically significant improvements by MDBO, supporting its efficacy over existing methods.

**Table 4:** Wilcoxon Rank Sum Test Results for Standard Benchmark Functions.

Fn	DBO	SCSO	AVOA	SCA	HHO	GWO	LCA	ZOA	WOA
F1	7.5E-10	7.5E-10	7.5E-10	7.5E-10	7.5E-10	7.5E-10	7.5E-10	7.5E-10	7.5E-10
F2	1.4E-09	1.4E-09	1.4E-09	1.4E-09	1.4E-09	1.4E-09	1.4E-09	1.4E-09	1.4E-09
F3	9.7E-11	9.7E-11	9.7E-11	9.7E-11	9.7E-11	9.7E-11	9.7E-11	9.7E-11	9.7E-11
F4	1.4E-09	1.4E-09	1.4E-09	1.4E-09	1.4E-09	1.4E-09	1.4E-09	1.4E-09	1.4E-09
F5	1.4E-09	1.4E-09	3.4E-01	1.4E-09	1.1E-06	1.4E-09	1.8E-09	1.4E-09	1.4E-09
F6	1.4E-09	1.4E-09	5.5E-02	1.4E-09	1.9E-03	1.4E-09	1.4E-09	1.4E-09	1.4E-09
F7	4.0E-08	1.4E-01	2.4E-02	1.4E-09	1.4E-01	1.4E-09	5.6E-06	6.1E-01	1.3E-08
F8	1.4E-09	1.4E-09	1.4E-09	1.4E-09	1.4E-09	1.4E-09	1.4E-09	1.4E-09	1.4E-09
F9	NaN	NaN	NaN	9.7E-11	NaN	9.7E-11	9.7E-11	NaN	3.4E-01
F10	NaN	NaN	NaN	9.7E-11	NaN	9.7E-11	9.7E-11	NaN	1.8E-09
F11	NaN	NaN	NaN	9.7E-11	NaN	9.7E-11	9.7E-11	NaN	1.6E-01
F12	1.4E-09	1.4E-09	7.0E-01	1.4E-09	2.4E-06	1.4E-09	1.4E-09	1.4E-09	1.4E-09
F13	1.4E-09	1.4E-09	4.5E-01	1.4E-09	3.6E-08	1.4E-09	1.4E-09	1.4E-09	1.4E-09
F14	4.4E-07	1.4E-10	8.3E-10	1.4E-10	1.4E-10	1.4E-10	1.4E-10	1.6E-10	1.4E-10
F15	1.6E-09	1.8E-09	1.6E-09	1.4E-09	1.4E-09	1.4E-09	1.4E-09	1.6E-09	1.4E-09
F16	1.2E-02	5.4E-10	3.8E-07	5.4E-10	5.4E-10	5.4E-10	5.4E-10	1.1E-08	5.4E-10
F17	NaN	9.7E-11	3.4E-01	9.7E-11	9.7E-11	9.7E-11	9.7E-11	4.0E-07	9.7E-11
F18	1.6E-02	1.3E-09	1.3E-09	1.3E-09	1.3E-09	1.3E-09	1.3E-09	1.4E-05	1.3E-09
F19	1.8E-04	1.4E-10	1.4E-10	1.4E-10	1.4E-10	1.4E-10	1.4E-10	1.4E-10	1.4E-10
F20	1.9E-01	4.1E-05	4.0E-02	7.7E-10	3.8E-09	1.2E-01	7.7E-10	3.1E-01	2.1E-02
F21	7.4E-06	3.8E-10	3.8E-10	3.8E-10	3.8E-10	3.8E-10	3.8E-10	3.8E-10	3.8E-10
F22	9.2E-07	2.5E-10	2.5E-10	2.5E-10	2.5E-10	2.5E-10	2.5E-10	2.5E-10	2.5E-10
F23	1.7E-07	7.7E-10	7.7E-10	7.7E-10	7.7E-10	7.7E-10	7.7E-10	7.7E-10	7.7E-10

**Table 5:** Wilcoxon Rank Sum Test Results for CEC-2019 Benchmark Suite.

Fn	SCSO	AVOA	SCA	HHO	GWO	LCA	ZOA	WOA
CEC01	5.85E-09	4.00E-08	1.04E-08	1.42E-09	1.60E-09	1.42E-09	1.42E-09	1.01E-06
CEC02	0.00129	3.13E-10	3.13E-10	3.13E-10	3.13E-10	3.13E-10	3.13E-10	3.13E-10
CEC03	0.716876	6.42E-10	0.000246	5.66E-10	5.66E-10	5.66E-10	5.66E-10	1.06E-09
CEC04	3.02E-07	5.85E-09	6.57E-09	1.42E-09	1.42E-09	6.89E-08	1.42E-09	1.42E-09
CEC05	0.000211	3.58E-08	0.000211	1.42E-09	1.42E-09	5.44E-05	1.42E-09	2.29E-09
CEC06	0.449223	1.64E-08	1.42E-09	2.78E-05	0.000227	1.38E-05	2.57E-08	5.85E-09
CEC07	0.050032	0.125319	0.013733	1.81E-06	0.277231	0.14561	1.60E-09	0.01613
CEC08	0.004614	0.547515	0.059826	1.49E-06	0.000357	0.712386	1.42E-09	0.091402
CEC09	1.84E-08	1.42E-09	7.38E-09	1.42E-09	1.42E-09	1.42E-09	1.42E-09	1.42E-09
CEC10	0.001367	5.12E-06	2.57E-09	0.002035	0.080766	0.000907	2.57E-09	2.87E-08

#### Friedman mean rank test

**Fig. 6:**
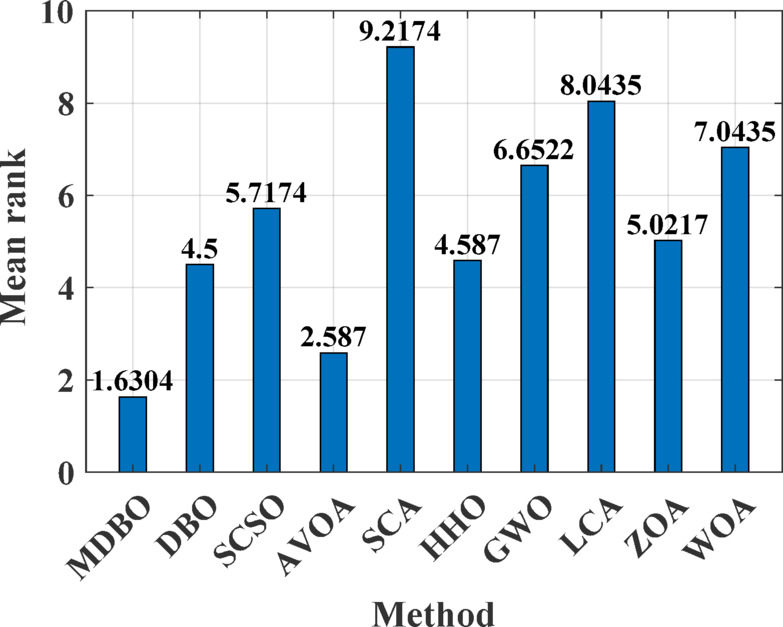
Friedman mean rank test results for 23 standard benchmark functions.

**Fig. 7:**
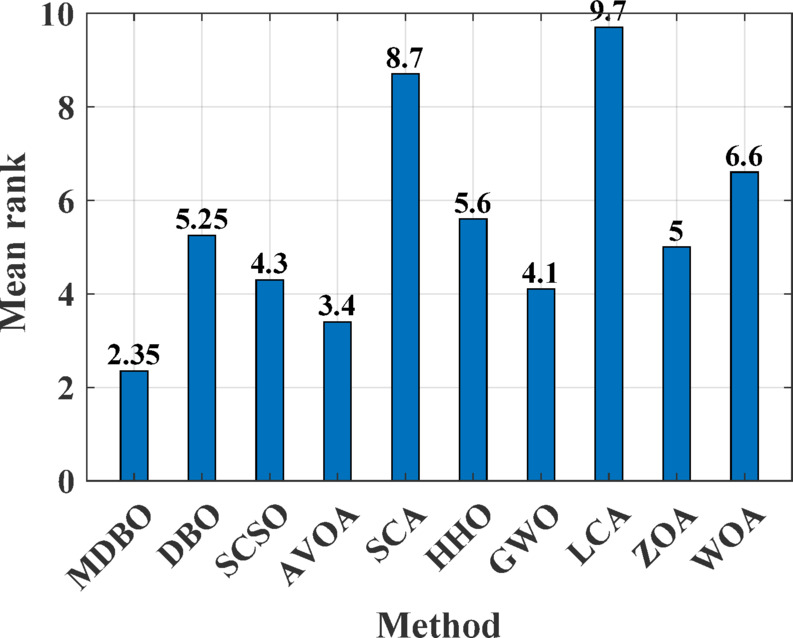
Friedman mean rank test results for CEC-2019 benchmark suite.

The Friedman mean rank test serves as a non-parametric statistical tool designed to assess the significance of differences among several optimization algorithms by evaluating their average rankings across a set of benchmark functions. Figures 6 and 7 illustrate the Friedman mean rank values obtained from two distinct benchmark suites. The results demonstrate that the proposed MDBO consistently secures the lowest mean rank, reflecting its superior performance relative to contemporary optimization methods. In contrast, the Sine Cosine Algorithm (SCA) exhibits the highest mean rank, indicating the least favorable performance within this comparative framework.

### Application of MDBO for different CHPED test systems

This section focuses on applying the proposed MDBO to solve four CHPED test systems: 4-unit, 7-unit, 24-unit, and 48-unit configurations. Table A.1 in Appendix A presents the specifications of the studied systems. The simulation setup (including the number of agents, iterations, and runs) remains consistent across all test systems for a fair comparison, following the parameters listed in Table 1.

#### Test system-1 (4-unit system)

**Fig. 8:**
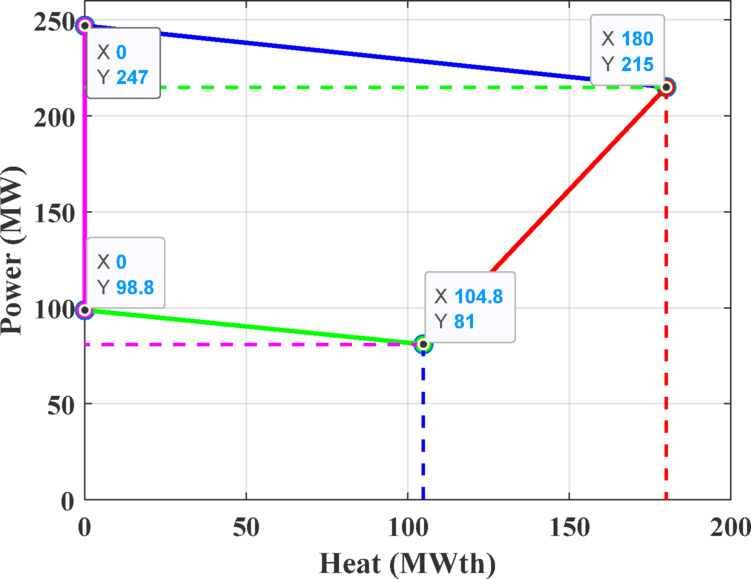
Feasible region for CHP Unit 2 in Test System-1.

**Fig. 9:**
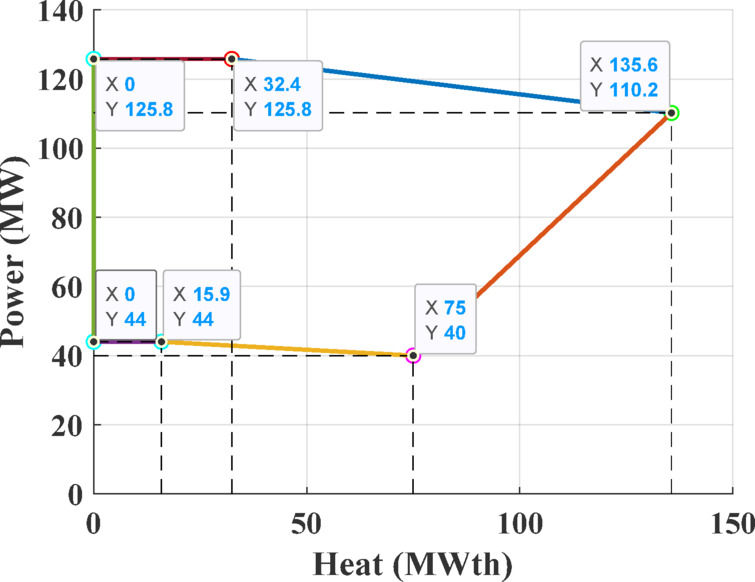
Feasible region for CHP Unit 3 in Test System-1.

The first test system consists of four generating units, including a single power-only unit, two CHP units, and one heat-only unit. This configuration is required to satisfy an electrical demand of 200 MW and a thermal demand of 115 MWth. The parameters defining the cost functions are details are taken from ^71^, while the permissible operating regions for the CHP units are depicted in Figs. 8 and 9.

**Fig. 10:**
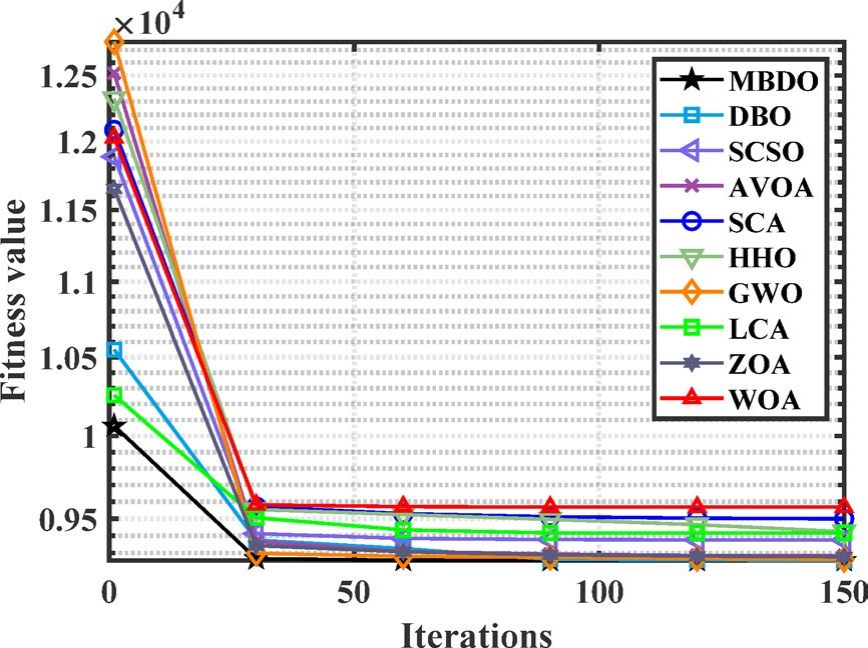
Convergence behavior of various optimizers for the 4-unit system.

**Fig. 11:**
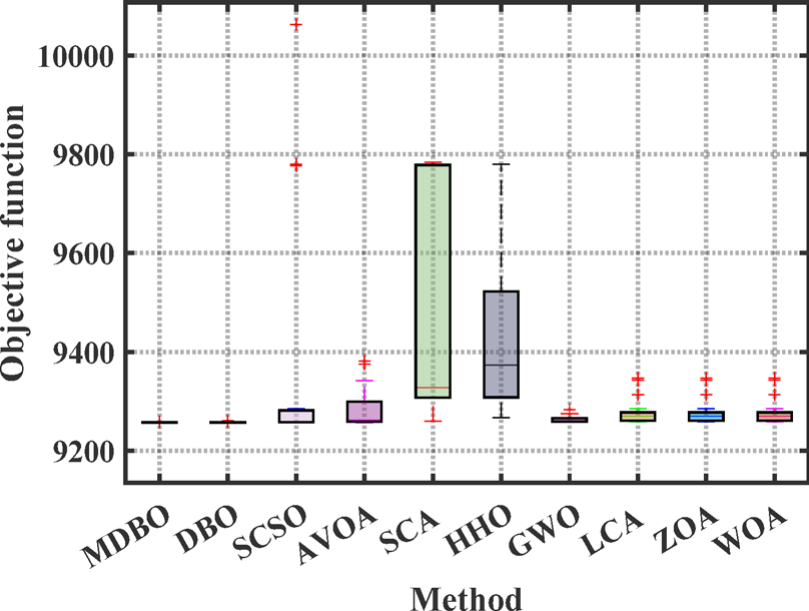
Boxplot analysis of optimization results for the 4-unit system.

In this configuration, power losses (Ploss), VPLE, and POZs are not considered. The aim is to minimize the whole operational cost without losses. Simulations were conducted using MDBO and other optimization methods. Table 6 presents the statistical outcomes (best, average, worst) for the 4-unit system, where the best average and optimal response are attained by the MDBO to decrease the entire cost of the system. Indeed, Table 7 provides comparative performance results. Figures 10 and 11 show the convergence trends and boxplot comparisons, where the efficient and stable response was observed by the proposed MDBO.

**Table 6:** Statistical performance results for 4-unit system (without considering power losses).

Solution optimizer	Average cost ($)	Best cost ($)	Worst cost ($)
MDBO	9257.2	9257.1	9258.7
DBO	9257.7	9257.1	9260.5
SCSO	9374.9	9257.2	10,063.0
AVOA	9285.4	9257.1	9381.6
SCA	9499.2	9259.4	9783.3
HHO	9424.0	9266.3	9779.8
GWO	9262.9	9257.6	9283.0
LLCA	9416.5	9304.0	9534.6
ZOA	9278.5	9258.5	9347.0
WOA	9568.3	9274.9	10,063.0

**Table 7:** Comparative analysis of the optimizers for 4-unit systems studied (without considering power losses).

Algorithm	Best cost ($)	Algorithm	Best cost ($)
RGA ^72^	9263.28	GA2^73^	9452.2
ARO ^31^	9257.198	GA1^73^	9267.2
		ACSA ^74^	9452.2

According to Tables 6 and 7, the lowest operational cost achieved by the MDBO was $9,257.1, outperforming (lower than) other algorithms by varying ratios: SCSO (0.0011%), SCA (0.0248%), HHO (0.0992%), GWO (0.0054%), LCA (0.504%), ZOA (0.0151%), WOA (0.1919%), RGA (0.0667%), ARO (0.00106%), GA2 (2.064%), GA1 (0.1089%), ACSA (2.0641%). It is noted here that some results for this case, the values observed were identical because of the low population size and iteration, but overall, the response of the proposed optimizer is observed to be better than the other state-of-the-art techniques.

Table B.1 in Appendix B shows the optimal scheduling outcomes for this test case. The solutions obtained by MDBO adhered to all operational constraints, with no violations observed.

#### Test system-2 (7-unit system)

This system is composed of a total of seven generating units, comprising four power-only units, two CHP units, and one heat-only unit. It is tasked with fulfilling a power demand of 600 MW alongside a thermal demand of 150 MWth. This system is analyzed under three distinct operational scenarios:

- Case 1: CHPED with VPLE,
- Case 2: CHPED with VPLE and PLs,
- Case 3: CHPED with VPLE, PLs, and POZs.

The cost function parameters are taken from reference ^71^, and a comprehensive discussion of the findings from these three cases is provided in the upcoming subsections.

##### CASE-1: CHPED with VPLE

In this case, only the VPLE was incorporated into the CHPED formulation. Simulations were performed using the MDBO and several comparative optimization algorithms under the same parameter settings. The statistical performance results are reported in Table 8. MDBO achieves the most cost-effective and consistent outcomes.

**Table 8:** Statistical results for the 7-unit system considering VPLE.

Solution optimizer	Average cost ($)	Best cost ($)	Worst cost ($)
MDBO	10,106.39	10,091.92	10,148.78
DBO	10,216.44	10,142.8	10,535.37
SCSO	10,231.64	10,116.87	10,478.09
AVOA	10,170.35	10,117.2	10,220.35
SCA	10,403.21	10,214.9	10,778.62
HHO	10,290.42	10,182.6	10,550.16
GWO	10,160.88	10,104.73	10,187.47
LLCA	10,229.78	10,112.03	10,344.03
ZOA	10,205.63	10,144.24	10,354.07
WOA	10,348.81	10,155.41	10,804.92

**Fig. 12:**
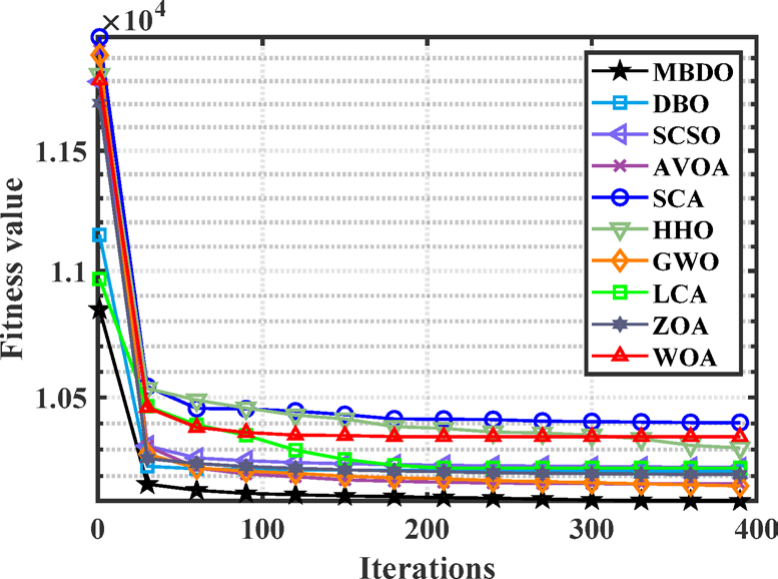
Convergence behavior of different optimizers for 7-unit system with VPLE.

**Fig. 13:**
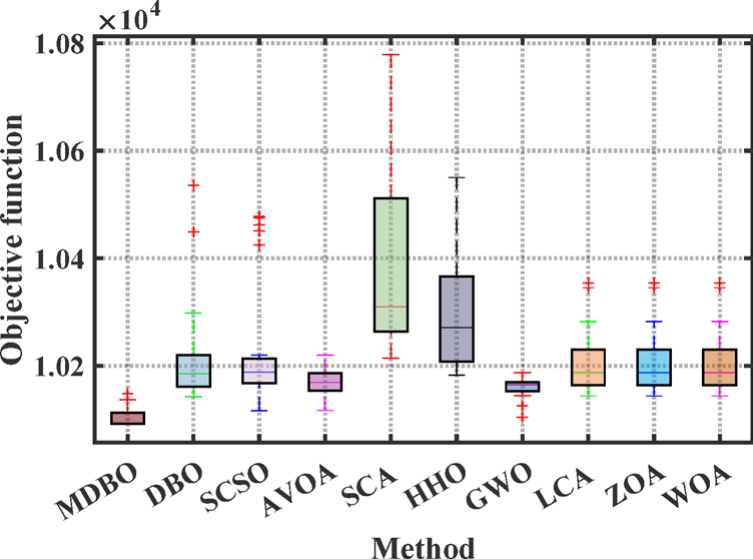
Boxplot analysis for cost values of different optimizers for 7-unit system with VPLE.

Figures 12 and 13 illustrate the convergence curves and boxplot responses obtained by various optimizers for minimizing the cost function. These figures demonstrate that the MDBO exhibits faster convergence and lower variability, indicating a more reliable and stable solution trajectory compared to the competing methods.

According to Tables 8 and 9, the MDBO achieved the lowest total generation cost of $10,091.92, outperforming several state-of-the-art optimizers. Specifically, the cost obtained by MDBO was lower than those of DBO by 0.502%, SCSO by 0.247%, AVOA by 0.249%, SCA by 1.204%, HHO by 0.890%, GWO by 0.127%, LLCA and LCA by 0.199%, ZOA by 0.516%, WOA by 0.625%, MRFO by 0.0041%, and ARO by 0.3051%. These results clearly demonstrate MDBO’s superior performance in minimizing total generation cost under the system conditions considered.

**Table 9:** Comparative cost performance across various algorithms for the 7-units system considering VPLE.

Algorithm	Best cost ($)	Algorithm	Best cost ($)
MDBO	10,091.92	LCA ^75^	10,112.03
DBO	10,142.8	MRFO^76^	10,092.33
ARO^31^	10,095	CFDBSDO^77^	10,091.92760

Table C.1 in Appendix C presents the optimal CHPED scheduling for the studied 7-unit system, considering the VPLE. The optimal schedule further validates MDBO’s robustness in addressing the nonlinearities and constraints introduced by VPLE, highlighting its potential for practical deployment in complex CHPED problems.

##### CASE-2: CHPED with VPLE and PL

In the second case of Test System-2, the VPLE and transmission PLs were both considered. This scenario adds complexity by considering real power losses, making it more representative of practical CHPED applications. To ensure a consistent comparison, the same simulation parameters were applied across all algorithms. The statistical results of various optimization techniques, including the proposed MDBO, are presented in Table 10. Among the examined algorithms, MDBO consistently produced the best cost outcomes with faster convergence and a more reliable distribution of results.

**Table 10:** Statistical results of the 7-unit system considering VPLE and PLs.

Solution optimizer	Average cost ($)	Best cost ($)	Worst cost ($)
MDBO	10,108.4	10,094.21	10,149.53
DBO	10,162.48	10,114.65	10,277.37
SCSO	10,203.96	10,118.25	10,428.81
AVOA	10,166.95	10,106.78	10,217.41
SCA	10,346.05	10,216.52	10,777.63
HHO	10,249.87	10,135.08	10,454.46
GWO	10,156.61	10,115.66	10,194.18
LLCA	10,231.12	10,131.39	10,320.16
ZOA	10,193.5	10,135.32	10,279.03
WOA	10,338.65	10,171.64	10,811.91

**Fig. 14:**
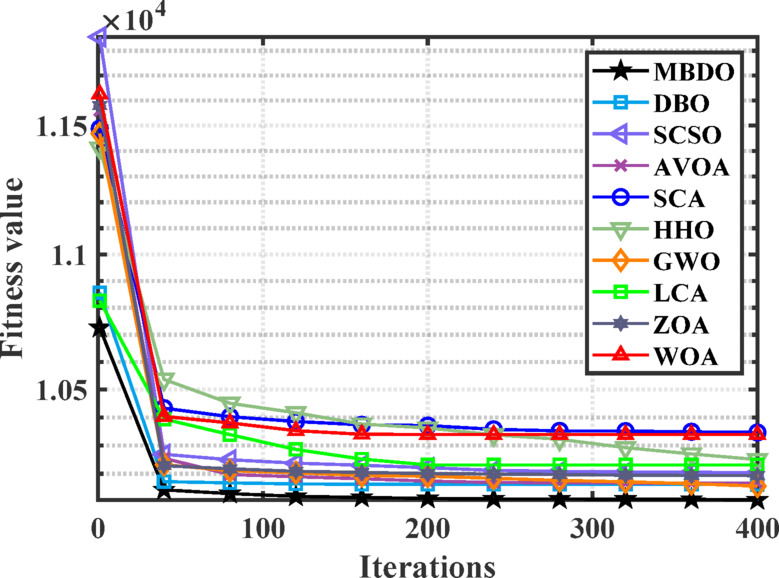
Convergence behavior of cost by different optimizers for the 7-unit system with VPLE and PLs.

**Fig. 15:**
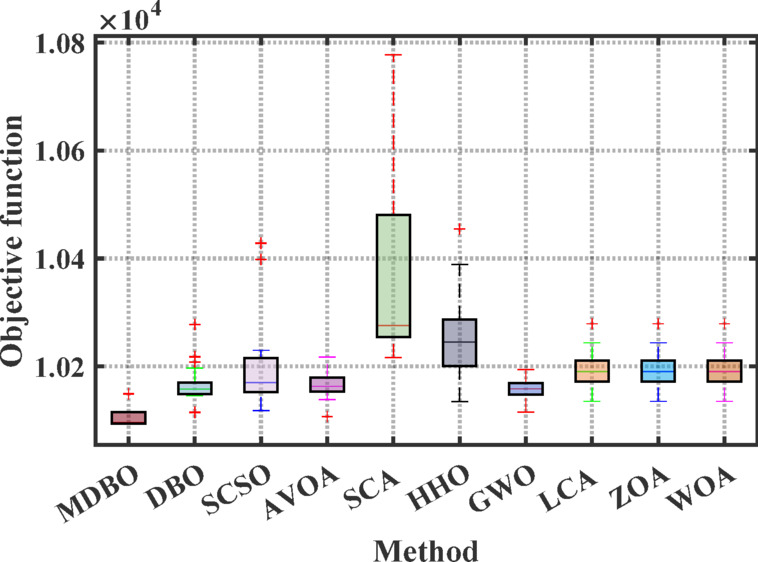
Boxplot comparison for different optimizers for the 7-unit system with VPLE and PLs.

Figures 14 and 15 illustrate the convergence behavior and boxplot distributions of the cost function achieved by various optimization algorithms considering VPLEs and PLs constraints. The proposed MDBO demonstrated both efficient and stable performance, achieving the lowest total system cost. As illustrated in these figures, the MDBO algorithm demonstrated a notably faster convergence rate relative to the other methods while simultaneously preserving a high degree of solution consistency, as evidenced by the diminished variability observed over multiple execution runs.

According to Tables 10 and 11, the minimum cost achieved by MDBO was $10,215.61, which was superior to those attained by the other optimizers. The corresponding percentage differences were: DBO (0.504%), SCSO (0.210%), AVOA (0.210%), SCA (1.118%), HHO (0.707%), GWO (0.0805%), LCA (0.167%), ZOA (0.488%), WOA (0.549%), MRFO (0.0037%), ARO (0.2806%).

**Table 11:** Comparative performance of MDBO and other optimizers for 7-unit system VPLE and PLs.

Algorithm	Best cost ($)	Algorithm	Best cost ($)
MDBO	10,094.21	BCO^78^	10,317
DBO	10,114.65	AIS^79^	10,355
IGA-NCM^14^	10,107.9071	CPSO^16^	10,325.3339
EP^80^	10,390	EMA^81^	10,111.0732
PSO^80^	10,613	ARO^31^	10,103.54

Table D1 in Appendix D shows the optimal CHPED scheduling for the studied 7-unit system, considering both VPLEs and PLs. The table compares results from various optimization algorithms, highlighting how the proposed MDBO performs in comparison to others. The results under this scenario highlight the robustness and adaptability of MDBO in handling the complex constraints of the CHPED model, effectively addressing both VPLE and PLs simultaneously.

##### CASE-3: CHPED with VPLE, PLs, and POZs

The third and most complex case incorporates all major nonlinearities, VPLEs, PLs, and POZs, into the CHPED model, resulting in a highly constrained and realistic optimization scenario. The transmission loss coefficients (B-matrix) used are the same as those in Case 2 of Test System 3, as defined in Eq. (21), while the parameters for the POZs are details taken from the reference ^71^.

The inclusion of POZs introduces non-convex constraints, which can lead traditional optimization algorithms to suffer from premature convergence or entrapment in local optima. This case, therefore, provides a comprehensive benchmark for evaluating the robustness and effectiveness of the MDBO in handling real-world operational complexities. The optimization performance of MDBO was benchmarked against several state-of-the-art algorithms, with statistical comparisons presented in Table 12. MDBO successfully achieved both the lowest average fuel cost and the best optimal solution, demonstrating its superior capability in addressing the challenges posed by this highly constrained CHPED problem.

**Table 12:** Statistical results for the 7-unit system considering VPLEs, PLs, and POZs.

Solution optimizer	Average cost ($)	Best cost ($)	Worst cost ($)
MDBO	10,144.56	10,101.28	10,207.4
DBO	10,220.71	10,146.48	10,308.7
SCSO	10,254.5	10,152.82	10,772.88
AVOA	10,189.78	10,154.36	10,221.12
SCA	10,440.9	10,223.74	10,815.75
HHO	10,284.4	10,165.7	10,521.88
GWO	10,181.46	10,117.46	10,391.26
LLCA	10,312.37	10,219.24	10,509.35
ZOA	10,205.93	10,160.28	10,263.52
WOA	10,484.31	10,181.12	10,811.92

**Fig. 16:**
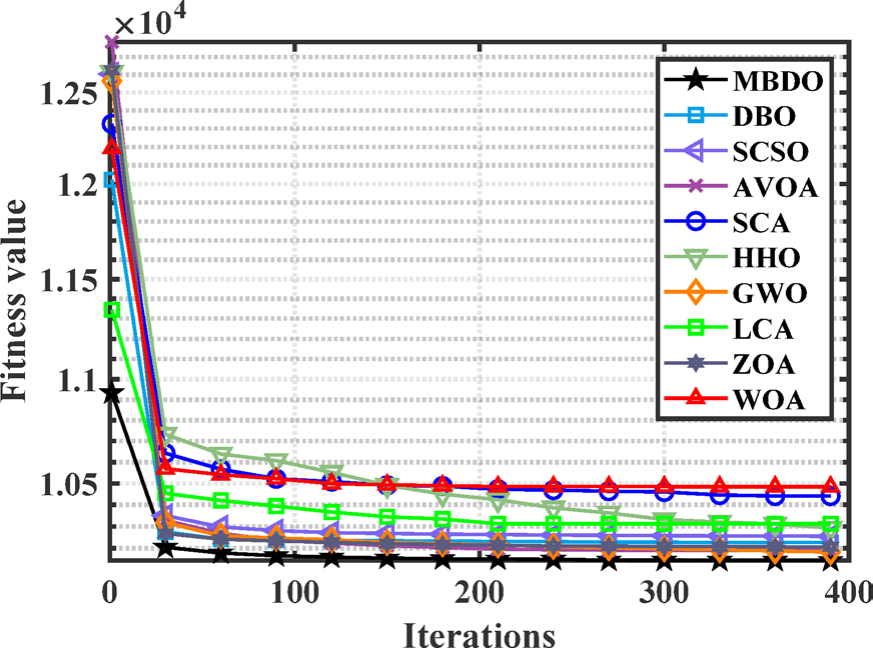
Convergence trend for the 7-unit system with VPLEs, PLs, and POZs.

**Fig. 17:**
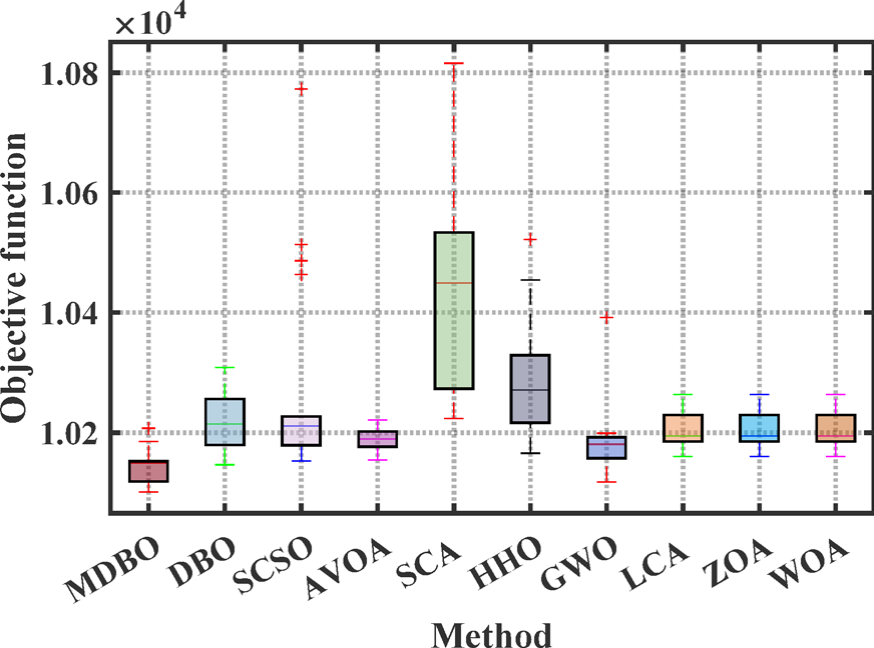
Boxplot of results of the 7-unit system with VPLEs, PLs, and POZs.

Figures 16 and 17 illustrate the convergence behavior and boxplot distributions of the cost minimization results obtained by MDBO and other studied benchmark optimizers for the CHPED system incorporating VPLEs, PLs, and POZs. The proposed MDBO demonstrates both efficiency and stability, consistently achieving lower cost values in this highly constrained scenario. As shown in these figures, MDBO exhibits rapid convergence and strong reliability, even in the presence of complex non-linear and non-convex constraints, further validating its robustness and effectiveness in solving real-world CHPED problems.

As shown in Tables 12 and 13, the cost obtained using MDBO was 10,101.28$, outperforming all other techniques with cost differences as follows: DBO (0.445%), SCSO (0.508%), AVOA (0.523%), SCA (1.198%), HHO (0.634%), GWO (0.159%), LCA (1.154%), ZOA (0.581%), WOA (0.784%), HTS (0.0296%), BLPSO (0.00028%), NDE (0.0324%), ARO (0.139%), GSO (0.0007%), and IGA-NCM (0.134%).

**Table 13:** Comparative performance of MDBO against others studied optimization techniques for the 7-unit system considering VPLEs, PLs, and POZs.

Algorithm	Best cost ($)	Algorithm	Best cost ($)
MDBO	10,101.28	NDE^82^	10,104.5561
DBO	10,146.48	ARO^31^	10,115.37
HTS^83^	10,104.2707	GSO^84^	10,101.3483
BLPSO^3^	10,101.3079	IGA-NCM^14^	10,114.841

Table E1 in Appendix E presents the optimal CHPED scheduling for the studied 7-unit system, considering VPLEs, PLs, and POZs. The table compares the results obtained using several optimization algorithms, including the proposed MDBO, to clarify the performance differences among these algorithms. It is important to note that, across all three cases of Test System 2 (as detailed in Tables C.1, D.1, and E.1), no constraint violations were observed. All system requirements were fully satisfied, confirming the validity of the solutions obtained. This demonstrates the reliability of MDBO in effectively handling complex, multi-constrained CHPED problems, reinforcing its suitability for real-world applications.

#### Test system-3 (24-unit system)

**Fig. 18:**
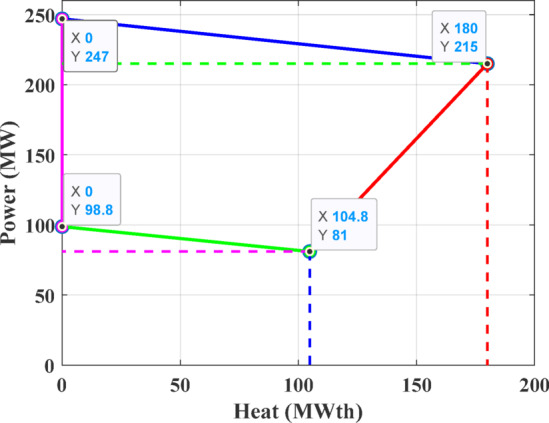
Feasibility limits for CHP units 14,16 in Test System-3.

**Fig. 19:**
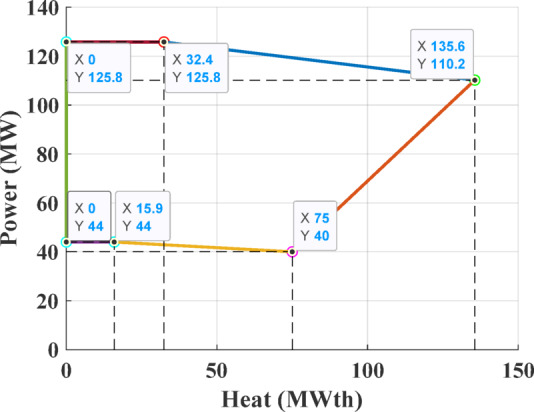
Feasibility limits for CHP units 15,17, in Test System-3.

**Fig. 20:**
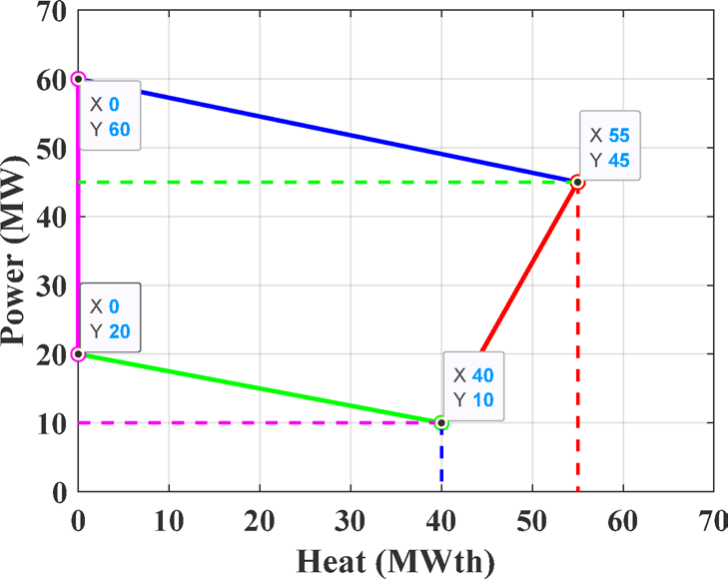
Feasibility region for CHP unit 18 in Test System-3.

**Fig. 21:**
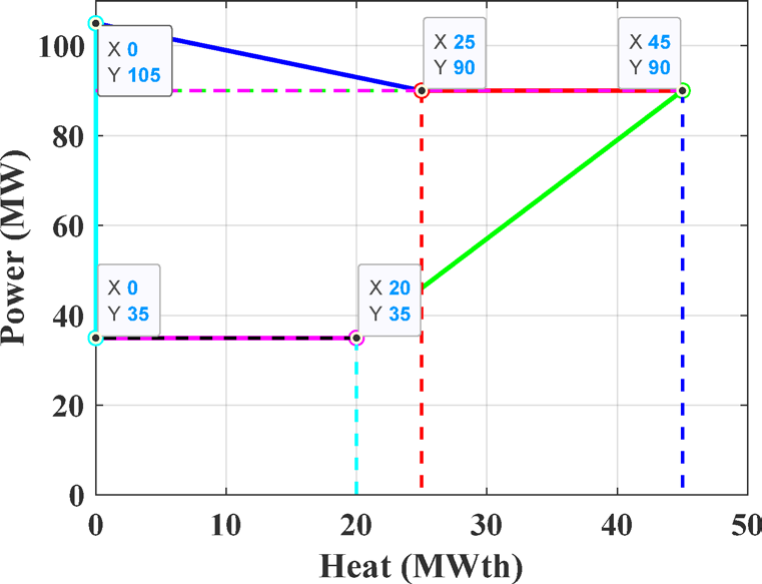
Feasibility region for CHP unit 19, in Test System-3.

The third test system consists of a CHPED system comprising 24 generating units, which include 13 power-only units, 6 CHP units, and 5 heat-only units. The system is required to meet 2,350 MW of electrical power and 1,250 MWth of thermal demand. The cost coefficients and operational limits for this system are details taken from reference ^71^, while the feasibility boundaries of CHP units are depicted in Figs. 18–21. The objective of this test case is to minimize the total operating cost, with cost functions that account for power transmission losses.

The simulations for this large-scale CHPED case were conducted using the input parameters specified in Table 1, applying various optimization algorithms, including the proposed MDBO. This complex test case includes PLs, making it a challenging benchmark for assessing the scalability and robustness of optimization techniques. The statistical results of each optimizer, comprising the average, best, and worst cost values, are summarized in Table 14. As shown, the proposed MDBO achieved the lowest average and optimal cost, demonstrating its superior performance.

**Table 14:** Statistical results of the 24-unit system with considering PLs.

Solution optimizer	Average cost ($)	Best cost ($)	Worst cost ($)
MDBO	58,244.74	57,803.47	58,725.33
DBO	59,140.23	58,238.78	60,671.92
SCSO	60,915.27	58,986.86	63,736.79
AVOA	59,578.07	58,560.10	62,499.07
SCA	66,885.64	63,253.40	69,785.73
HHO	60,373.13	59,170.30	62,965.82
GWO	58,388.17	58,000.72	60,715.86
LLCA	62,586.94	60,972.99	64,181.70
ZOA	60,530.11	59,436.98	61,854.82
WOA	63,620.08	59,861.57	68,465.08

**Fig. 22:**
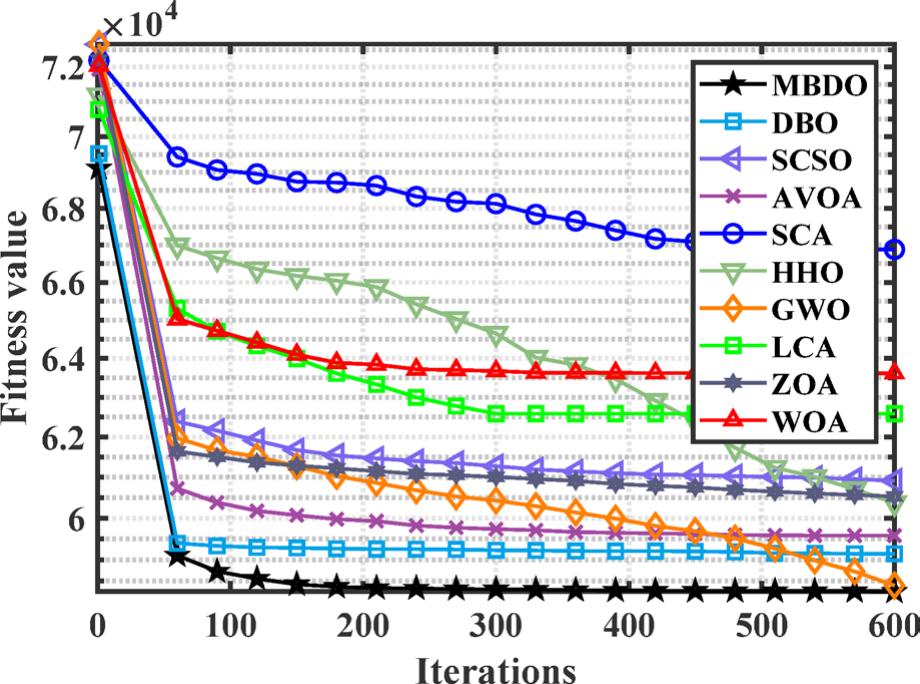
Convergence behavior of cost by different optimizers for the 24-unit system considering PLs.

**Fig. 23:**
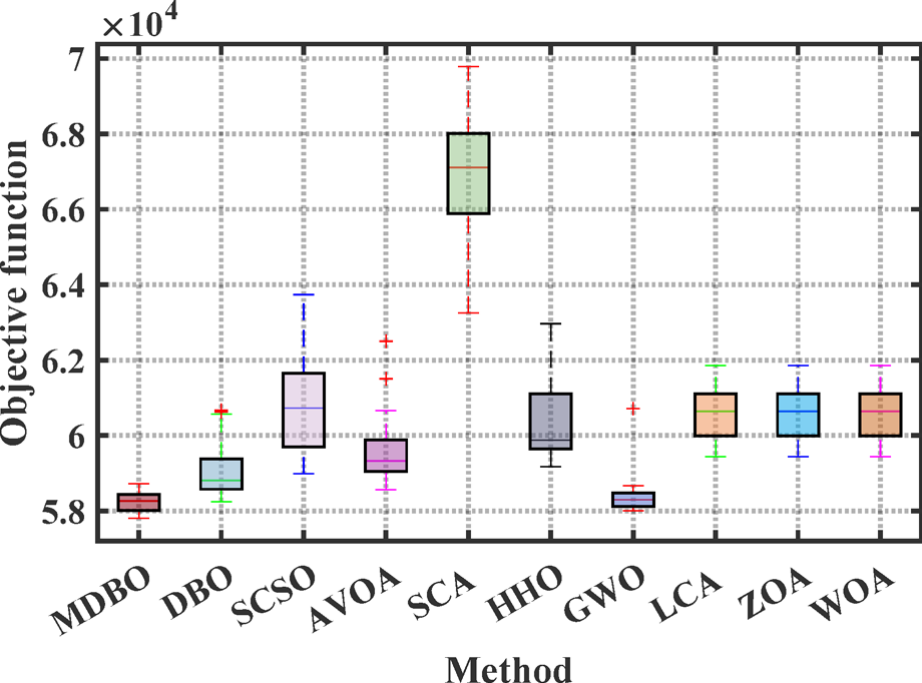
Boxplot of results of the 24-unit system considering PLs.

Figures 22 and 23 illustrate the convergence trajectories and boxplot representations of the outcomes produced by the different optimization algorithms under study. The proposed MDBO algorithm exhibited not only accelerated convergence but also robust and stable performance, consistently attaining the optimal solution for the CHPED problem.

The results presented in Table 14 underscore the superior performance of the proposed MDBO algorithm, which achieved the lowest and best and average cost values among all evaluated optimizers. This outcome highlights the effectiveness of MDBO in solving large-scale, multivariable CHPED problems with nonlinear constraints. According to Tables 14 and 15, MDBO achieved a total cost of $57,803.47, outperforming all other compared algorithms. The percentage differences in cost relative to MDBO are as follows: DBO (0.747%), SCSO (2.006%), AVOA (1.292%), SCA (8.616%), HHO (2.309%), GWO (0.340%), LLCA (5.198%), ZOA (2.748%), WOA (3.438%), TLBO (0.351%), OTLBO (0.091%), RCGA-IMM (0.453%), ARO (0.104%), MGSO (0.033%), SGWO (0.419%), HBOA (0.329%), SNS (0.161%), OGSO (0.044%), GSA (0.548%), EMA (0.038%), GSO (0.549%), CPSO (3.235%), TVAC-PSO (0.548%), AFDB-ARO (0.0382%), IGA-NCM (0.391%), ACS-DEM (0.163%), IHT (0.259%), NDE (0.0427%), and IGSO (0.423%). These results demonstrate MDBO’s outstanding capability in delivering cost-effective and reliable solutions for complex CHPED systems.

**Table 15:** Statistical comparison of MDBO results with the other state-of-the-art techniques for 24-unit system with PLs.

Algorithm	Best cost ($)	Algorithm	Best cost ($)
MDBO	57,803.47	GSA^85^	58,121.86
DBO	58,238.78	EMA ^13^	57,825.48
TLBO ^16^	58,007	GSO^26^	58,122.71
OTLBO^16^	57,856.27	CPSO ^86^	59,736.26
RCGA-IMM^87^	58,066.63	TVAC-PSO ^86^	58,122.75
ARO ^31^	57,863.78	AFDB-ARO ^31^	57,825.54
MGSO ^26^	57,822.83	IGA-NCM ^88^	57,826.09
SGWO* ^89^	57,827.71	ACS-DEM ^90^	57,897.61
HBOA ^2^	57,994.51	IHT ^91^	57,953.53
SNS ^92^	57,896.63	NDE*^82^	57,828.19
OGSO ^93^	57,829.24	IGSO ^94^	58,049.02

(*P*_*d*_ = 2350 MW and *H*_*d*_ = 1250 MWth)

The optimal CHPED schedules, accounting for power losses, were obtained using the various studied optimization algorithms, including the proposed MDBO, and are presented in Table 16. During the solution process for this large-scale CHPED system, no constraint violations were observed, and all operational limits were strictly adhered to. These results confirm the feasibility, accuracy, and robustness of all optimization methods evaluated, particularly the MDBO, which demonstrated high reliability in producing constraint-compliant solutions under complex system conditions.

**Table 16:** Optimal CHPED scheduling for the 24-unit system considering power losses.

Unit	MDBO	DBO	SCSO	AVOA	SCA	HHO	GWO	LLCA	ZOA	WOA
$${P}_{1}$$	448.83	448.80	448.80	359.04	640.85	269.28	628.89	548.21	448.95	538.56
$${P}_{2}$$	299.99	300.00	299.99	299.20	270.00	299.23	299.48	276.27	299.52	299.90
$${P}_{3}$$	299.20	304.23	227.68	139.58	0.00	224.35	73.06	226.63	224.63	295.13
$${P}_{4}$$	109.87	60.00	109.87	110.40	60.00	157.16	160.99	112.28	110.09	60.00
$${P}_{5}$$	159.79	180.00	65.12	173.50	72.01	173.64	110.01	95.57	68.71	60.00
$${P}_{6}$$	60.00	60.00	110.84	159.73	133.26	175.71	110.96	102.56	159.92	179.97
$${P}_{7}$$	159.81	159.73	60.46	159.82	61.46	76.17	110.37	83.92	128.37	179.97
$${P}_{8}$$	159.93	60.00	161.06	159.73	180.00	111.47	110.96	100.96	109.91	60.00
$${P}_{9}$$	110.00	60.00	164.24	107.87	102.42	64.60	159.55	109.34	115.94	60.00
$${P}_{10}$$	40.00	120.00	49.22	77.08	102.09	85.18	77.37	69.70	81.90	43.95
$${P}_{11}$$	40.00	120.00	114.56	47.76	88.12	114.13	41.74	68.92	66.64	119.98
$${P}_{12}$$	92.42	55.00	64.35	86.19	83.30	119.45	55.78	81.67	87.36	55.00
$${P}_{13}$$	55.00	120.00	92.40	119.99	63.02	93.43	99.18	72.38	72.41	63.07
$${P}_{14}$$	98.97	88.99	107.78	98.88	180.52	96.27	82.68	101.63	88.66	81.07
$${P}_{15}$$	40.21	40.00	57.12	71.33	78.47	68.75	40.87	70.63	53.38	40.00
$${P}_{16}$$	90.96	88.25	109.88	94.53	84.47	134.92	94.78	114.94	105.72	119.33
$${P}_{17}$$	40.00	40.00	42.13	40.38	71.06	40.09	47.55	56.35	55.15	40.00
$${P}_{18}$$	10.00	10.00	18.35	10.00	24.55	10.53	10.01	13.59	31.10	19.06
$${P}_{19}$$	35.00	35.00	46.14	35.00	54.41	35.65	35.76	44.44	41.64	35.00
$${h}_{14}$$	114.88	109.28	119.76	114.83	128.87	112.37	105.66	105.08	109.09	104.84
$${h}_{15}$$	75.18	75.00	89.77	102.05	94.07	99.77	75.74	97.25	86.47	74.51
$${h}_{16}$$	110.35	108.87	120.97	112.39	98.86	133.32	112.26	120.78	118.64	83.19
$${h}_{17}$$	74.96	75.00	76.79	75.33	91.02	75.06	81.32	77.39	88.05	74.12
$${h}_{18}$$	41.00	41.00	41.00	41.00	41.00	40.81	41.00	39.82	40.98	40.99
$${h}_{19}$$	23.20	23.20	23.15	23.20	18.77	23.11	23.20	22.61	21.43	19.35
$${h}_{20}$$	450.45	457.65	422.37	421.20	439.61	406.05	451.01	449.19	425.46	493.05
$${h}_{21}$$	60.00	60.00	60.00	60.00	60.00	60.00	59.81	55.06	59.98	59.99
$${h}_{22}$$	59.99	60.00	56.21	60.00	49.07	60.00	60.00	51.45	59.91	59.99
$${h}_{23}$$	120.00	120.00	119.98	120.00	108.73	120.00	120.00	116.71	120.00	119.98
$${h}_{24}$$	120.00	120.00	120.00	120.00	120.00	119.52	120.00	114.66	119.99	119.98
T.P (MW)	2350.00	2350.00	2350.00	2350.00	2350.00	2350.00	2350.00	2350.00	2350.00	2350.00
T.H MWth)	1250.00	1250.00	1250.00	1250.00	1250.00	1250.00	1250.00	1250.00	1250.00	1250.00
T.Cost ($)	57,803.47	58,238.78	58,986.86	58,560.10	63,253.40	59,170.30	58,000.72	60,972.99	59,436.98	59,861.57

#### Test system-4 (48-unit system)

The fourth studied test system consists of 48 generating units, including 26 power-only units, 12 CHP units, and 10 heat-only units. This configuration is required to satisfy an electrical demand of 4700 MW and a thermal demand of 2500 MWth. The parameters defining the cost functions are details are taken from ^71^. The objective of this test case is to minimize the total operating cost, with cost functions that account for VPLE and POZs. The simulations for this large-scale CHPED case were conducted and listed in Table 17, applying various optimization algorithms, including the proposed MDBO. This complex test case includes VPLE and POZs, making it a challenging benchmark for assessing the scalability and robustness of optimization techniques.

**Table 17:** Optimal CHPED scheduling for the 48-unit system considering VPLE and POZs.

Unit	MDBO	DBO	SCSO	AVOA	SCA	HHO		GWO	LCA	ZOA	WOA
P1	628.8	677.0	359.2	89.8	106.5	636.9		630.3	686.4	628.3	538.6
P2	300.1	360.0	291.7	360.0	360.0	272.5		360.0	343.2	299.3	270.0
P3	299.4	0.0	299.8	299.3	134.7	2.0		90.5	240.9	76.9	282.0
P4	159.9	180.0	109.4	88.0	69.5	141.6		160.1	104.8	159.8	136.8
P5	113.5	180.0	66.9	153.7	170.4	107.7		63.8	70.1	81.3	78.8
P6	60.0	60.0	103.0	158.8	170.5	143.3		71.9	126.1	95.6	121.6
P7	61.0	60.0	148.3	161.6	74.6	126.7		67.3	121.8	109.8	136.5
P8	111.9	60.0	63.3	80.4	69.5	157.5		110.1	97.8	75.5	136.7
P9	60.2	180.0	114.3	115.6	107.7	155.3		71.1	156.0	110.1	66.1
P10	115.9	40.0	115.3	60.7	58.4	94.4		115.5	58.6	64.0	111.9
P11	118.3	114.7	113.4	40.4	111.0	75.7		116.0	75.8	117.6	77.4
P12	93.0	55.0	99.0	111.9	73.8	83.3		114.0	82.0	120.0	60.7
P13	92.2	120.0	108.1	60.9	106.2	102.7		101.2	84.8	93.7	86.0
P14	90.1	0.0	629.1	260.0	519.7	3.2		629.0	102.4	179.6	23.7
P15	300.2	360.0	299.5	300.2	270.0	283.7		299.8	284.5	360.0	270.0
P16	229.0	360.0	1.9	252.6	0.0	11.5		12.4	174.6	161.1	211.8
P17	162.9	60.0	159.7	89.2	103.3	161.7		71.1	106.1	110.1	95.8
P18	161.4	60.0	72.1	98.5	60.0	173.5		62.7	85.5	63.7	99.0
P19	60.8	159.8	111.9	102.2	146.1	60.8		60.7	101.6	98.2	165.1
P20	111.1	180.0	66.4	160.1	83.2	175.9		75.6	73.2	114.1	144.3
P21	160.0	60.0	68.2	159.7	60.0	165.4		113.8	83.7	148.7	60.0
P22	60.6	110.3	166.1	67.4	145.7	114.5		87.4	115.5	118.4	60.0
P23	115.1	120.0	58.3	83.3	56.4	89.9		63.2	108.1	114.9	40.0
P24	119.7	120.0	102.7	114.8	59.2	103.0		80.3	114.1	63.9	104.4
P25	55.5	93.1	55.5	101.8	94.1	62.6		115.5	61.4	98.4	69.7
P26	94.6	55.0	90.8	99.2	96.6	109.4		93.0	58.2	92.7	55.2
P27	85.7	126.1	83.9	134.5	219.9	173.7		109.1	103.8	119.1	164.9
P28	40.0	105.3	63.4	45.3	85.6	51.5		42.3	107.5	41.6	76.7
P29	112.4	127.5	141.4	177.3	247.0	153.3		178.7	137.5	126.3	174.2
P30	40.7	40.0	40.5	40.3	70.1	81.0		44.1	59.0	69.8	89.0
P31	10.1	10.0	18.5	10.0	31.7	24.1		10.5	28.8	31.3	29.0
P32	73.3	72.7	41.1	35.4	92.5	75.4		75.3	62.6	80.1	35.0
P33	140.0	133.9	98.3	160.3	209.5	117.3		111.5	122.2	140.7	202.0
P34	50.0	51.7	41.3	86.6	120.8	74.2		40.7	103.7	53.8	40.0
P35	85.5	122.9	148.1	180.8	173.3	174.3		124.1	97.4	147.4	167.0
P36	40.1	40.0	47.6	74.4	47.0	74.1		40.0	65.0	66.3	89.8
P37	10.0	10.0	28.8	10.0	11.2	11.6		10.8	18.1	17.5	44.7
P38	77.1	35.0	73.1	75.0	84.2	74.8		76.8	77.0	50.4	85.4
H27	106.7	130.1	106.3	134.8	0.0	132.3		120.0	12.1	126.1	143.7
H28	74.6	131.1	95.1	79.5	74.3	57.1		76.8	104.1	76.3	73.9
H29	122.4	130.9	66.1	158.9	0.0	104.4		159.3	111.3	56.4	142.9
H30	75.5	75.0	75.3	75.2	0.0	108.6		78.0	86.6	100.5	117.1
H31	55.0	55.0	54.2	55.0	37.0	52.5		55.0	23.3	50.4	45.3
H32	17.0	17.0	45.0	45.0	18.4	17.1		17.8	17.7	20.0	42.2
H33	137.8	134.3	5.2	149.3	0.0	123.9		121.7	23.7	138.2	119.5
H34	83.5	85.1	2.8	115.3	5.0	102.8		1.5	56.9	86.9	41.9
H35	107.3	128.3	142.5	160.8	148.2	152.7		128.7	55.1	127.7	143.7
H36	74.4	75.0	81.5	104.7	0.0	99.5		51.8	37.4	49.8	101.1
H37	54.8	55.0	54.9	55.0	42.9	48.7		54.5	31.0	55.0	32.0
H38	19.0	45.0	17.2	18.2	17.0	17.5		18.9	26.8	44.0	36.3
H39	434.8	359.3	666.8	324.8	1073.1	285.6		543.1	834.3	510.5	439.7
H40	59.2	60.0	8.0	60.0	11.0	60.0		60.0	55.9	60.0	12.3
H41	60.0	60.0	11.2	60.0	0.0	54.7		9.1	37.6	52.1	50.6
H42	120.0	120.0	116.1	118.3	111.2	118.1		120.0	51.5	96.2	87.7
H43	120.0	120.0	119.4	119.6	0.0	112.3		119.9	35.4	76.3	97.1
H44	418.5	358.9	657.1	324.6	961.9	629.4		564.3	713.6	530.3	461.5
H45	59.5	60.0	44.7	60.0	0.0	50.3		19.7	38.2	30.8	33.1
H46	60.0	60.0	10.5	60.0	0.0	59.3		59.9	18.5	57.9	38.4
H47	120.0	120.0	120.0	102.5	0.0	13.7		0.0	17.8	119.9	120.0
H48	120.0	120.0	0.0	118.5	0.0	99.6		119.9	111.4	34.8	120.0
Total Power	4700.0	4700.0	4700.0	4700.0	4700.0	4700.0		4700.0	4700.0	4700.0	4700.0
Total Heat	2500.0	2500.0	2500.0	2500.0	2500.0	2500.0		2500.0	2500.0	2500.0	2500.0
Total Cost	117,952.4	119,056.2	136,977.4	121,649.5	192,611.6	131,558.8	129,846.2		153,210.1	128,399.7	132,830.8

The optimal CHPED schedules, accounting for VPLE and POZs, were obtained using the various studied optimization algorithms, including the proposed MDBO, and are presented in Table 17. During the solution process for this large-scale CHPED system, no constraint violations were observed, and all operational limits were strictly adhered to. These results confirm the feasibility, accuracy, and robustness of all optimization methods evaluated, particularly the MDBO, which demonstrated high reliability in producing constraint-compliant solutions under complex system conditions. Also, the presented results underscore the superior performance of the proposed MDBO algorithm, which achieved the lowest total cost value among all evaluated optimizers. This outcome highlights the effectiveness of MDBO in solving large-scale, multivariable CHPED problems with nonlinear constraints.

- Finally, according to the results obtained of the standard of the CEC-2019 benchmark functions the MDBO has the best performance compared to SCSA, AVOA, SCA, HHO, GWO, LCA, ZOA, and WOA in terms of the statistical results of Tables 2 and 3. Furthermore, the MDBO has stable and speed convergence characteristics compared to the comparative algorithms as depicted in Figs. 2 and 3.
- The MDBO is superior optimizer to solve the CHPED in which the minimum operating costs were obtained compared to the other optimization algorithms for the small, the medium and the large test systems. The minimum operation cost by MDBO for the 4-unit system is 9257.1 $. The minimum operation costs for 7-unit test system with VPLE is 10,091.92 $, the minimum cost with VPLE and PLs is 10,094.21 $ and, the minimum cost with VPLE, PLs, and POZs is 10,101.28 $. The minimum cost of 24-Unit System with 57,803.47 $. The minimum cost with 48-unit system is system considering VPLE and POZs is 117,952 $.

## Conclusions

This study critically examined the capability of the Modified Dung Beetle Optimizer (MDBO) in addressing the Combined Heat and Power Economic Dispatch (CHPED) problem, incorporating practical operational constraints such as transmission power losses (PLs), valve-point loading effects (VPLEs), and prohibited operating zones (POZs). The MDBO was developed to surmount common shortcomings of traditional metaheuristic algorithms, notably premature convergence and stagnation in the search process. By effectively enhancing the balance between exploration and exploitation phases, the MDBO demonstrated superior optimization performance. Its effectiveness was rigorously tested on four benchmark systems of CHPED problem of varying complexity: a 4-unit, a 7-unit, a 24-unit, and a 48-unit configuration. Extensive comparative analyses were performed against a broad spectrum of contemporary optimization algorithms, including Whale Optimization Algorithm (WOA), Sand Cat Swarm Optimization (SCSO), Zebra Optimization Algorithm (ZOA), African Vultures Optimization Algorithm (AVOA), Grey Wolf Optimizer (GWO), Harris Hawks Optimization (HHO), Liver Cancer Algorithm (LCA), and the original Dung Beetle Optimizer (DBO). The findings revealed that MDBO consistently outperformed these approaches by achieving lower total operational costs, accelerated convergence rates, and entirely feasible solutions across all test cases. Further, the robustness and scalability of MDBO were substantiated through evaluations using benchmark functions from the CEC-2019 suite, affirming its efficacy as a potent and dependable optimization method suitable for both small- and large-scale CHPED applications. In future work, incorporating the demand of electric vehicles and energy storage systems can be included in solving the CHPED problem.

## Supplementary Information

Below is the link to the electronic supplementary material.

Supplementary Material 1

## Data Availability

All data generated or analyzed during this study are included in this article.

## Abbreviations

AIS: Artificial immune system
ARO: Artificial rabbits’ optimization
BCO: Bee colony optimization
BLPSO: Biogeography-based learning particle swarm optimization.
CFDBSDO: Chaotic map-based fitness-distance balance supply–demand optimization algorithm
CHP: Combined Heat and Power
CHPED: Combined Heat and Power Economic Dispatch
DBO: Dung Beetle Optimizer
EMA: Exchange market algorithm
GA1: Genetic Algorithm 1
GA2: Genetic Algorithm 2
GSA: Gravitational search algorithm
GSO: Group search optimization
HTS: Heat transfer search algorithm
IACSA: Improved ant colony search algorithm
IGA-NCM: Improved genetic algorithm with novel crossover and mutation operators.
LCA: Line-up competition algorithm
MDBO: Modified Dung Beetle Optimizer
MGSO: Modified group search optimizer
MRFO: Manta ray foraging optimization algorithm
NDE: Niching differential evolution algorithm
OTLBO: Oppositional teaching learning-based optimization
PLs: Power Losses
POZs: Prohibited Operating Zones
RCGA-IMM: Real coded genetic algorithm with improved mühlenbein mutation
SPSO: Selective particle swarm optimization.
TLBO: Oppositional teaching learning-based optimization
TVAC-PSO: Time varying acceleration coefficients particle swarm optimization
VPLE: Valve-Point-Loading-Effect

## Code availability

The code is available from the corresponding author on reasonable request.
