# Supplementary material for: A modified Dung Beetle optimizer for combined heat and power economic dispatch considering power losses, valve-point effects, and operating constraints
Source: Sci Rep. 2026 Jan 16;16:2278. doi: 10.1038/s41598-025-32060-4 (PMC12816586; doi:10.1038/s41598-025-32060-4)
Supplement: Supplementary file 1 — Supplementary Material 1 [file 41598_2025_32060_MOESM1_ESM.docx]

**Appendix A**

**Table A.1**. Specifications of the studied test systems.

| **Test System** | **No. Units** | **Types of Units** | | | **Dimensions** | **Demands** | | **Case** | **Objective Function** | | |
| --- | --- | --- | --- | --- | --- | --- | --- | --- | --- | --- | --- |
|  |  | **Power only** | **CHP** | **Heat only** |  | **P_demand_ (MW)** | **H_demand_ (MWth)** |  | **P_Loss_** | **VPLF** | **POZs** |
| 1 | 4 | 1 | 2 | 1 | 6 | 200 | 115 | 1 | - | - | - |
| 2 | 7 | 4 | 2 | 1 | 9 | 600 | 150 | 1 | - | ✓ | - |
|  |  |  |  |  |  |  |  | 2 | ✓ | ✓ | - |
|  |  |  |  |  |  |  |  | 3 | ✓ | ✓ | ✓ |
| 3 | 24 | 13 | 6 | 5 | 30 | 2350 | 1250 | 1 | - | ✓ | ✓ |
| 4 | 48 | 26 | 12 | 10 | 60 | 4700 | 2500 | 1 | - | ✓ | ✓ |

**Appendix B**

**Table B.1**. Optimal CHPED schedule for the 4-unit system of the 4-unit system (*P_d_* = 200 MW and *H_d_* = 115 MWth).

| **Algorithm** | $P_{1}$ | $P_{2}$ | $P_{3}$ | $H_{2}$ | $H_{3}$ | $H_{4}$ |
| --- | --- | --- | --- | --- | --- | --- |
| MDBO | **0** | **160** | **40** | **40** | **74.99999** | **0.000001** |
| DBO | 0 | 160 | 40 | 40.00003 | 74.99997 | 0000002 |
| SCSO | 0 | 160 | 40 | 39.99698 | 74.99728 | 0.005733 |
| AVOA | 1.52E-08 | 160 | 40 | 40.00008 | 74.99992 | 4.41E-06 |
| SCA | 0 | 160 | 40 | 39.90039 | 74.94005 | 0.159562 |
| HHO | 3.25E-07 | 159.9301 | 40.06993 | 41.26391 | 73.73609 | 2.48E-07 |
| GWO | 0 | 160 | 40 | 40.0667 | 74.93078 | 0.002519 |
| LLCA | 0.025036 | 157.9097 | 42.06528 | 37.56408 | 75.60811 | 1.827807 |
| ZOA | 0.054855 | 159.9451 | 40 | 40.00019 | 74.99373 | 0.006074 |
| WOA | 0 | 160 | 40 | 42.69675 | 72.30296 | 0.000283 |

**Appendix C**

**Table C.1.** Optimal CHPED scheduling for the 7-unit system under VPLE (*P_d_* = 600 MW and *H_d_* = 150 MWth).

| **Unit** | **MDBO** | **DBO** | **SCSO** | **AVOA** | **SCA** | **HHO** | **GWO** | **LLCA** | **ZOA** | **WOA** |
| --- | --- | --- | --- | --- | --- | --- | --- | --- | --- | --- |
| $P_{1}$ | **44.937** | 35.166 | 37.802 | 43.253 | 10.000 | 10.791 | 42.434 | 45.701 | 48.404 | 32.459 |
| $P_{2}$ | **98.540** | 20.000 | 20.234 | 98.663 | 35.350 | 123.195 | 99.049 | 98.749 | 94.583 | 105.620 |
| $P_{3}$ | **112.67** | 30.000 | 112.997 | 30.085 | 30.703 | 33.602 | 112.897 | 113.041 | 30.001 | 30.192 |
| $P_{4}$ | **209.82** | 379.632 | 294.747 | 294.725 | 389.501 | 295.621 | 209.918 | 208.940 | 294.724 | 295.034 |
| $P_{5}$ | **94.034** | 95.203 | 94.220 | 93.274 | 94.447 | 96.792 | 95.702 | 93.459 | 92.288 | 96.464 |
| $P_{6}$ | **40.000** | 40.000 | 40.000 | 40.000 | 40.000 | 40.000 | 40.000 | 40.109 | 40.000 | 40.230 |
| $H_{5}$ | **28.062** | 21.180 | 27.032 | 32.538 | 29.878 | 12.054 | 18.488 | 31.463 | 38.363 | 13.752 |
| $H_{6}$ | **75.000** | 75.000 | 71.730 | 67.165 | 71.988 | 72.380 | 72.894 | 67.197 | 65.532 | 75.156 |
| $H_{7}$ | **46.938** | 53.820 | 51.239 | 50.298 | 48.134 | 65.567 | 58.618 | 51.341 | 46.105 | 61.093 |
| $P_{losses}$ | **600.00** | 600.000 | 600.000 | 600.000 | 600.000 | 600.000 | 600.000 | 600.000 | 600.000 | 600.000 |
| T.P (MW) | **150.00** | 150.000 | 150.000 | 150.000 | 150.000 | 150.000 | 150.000 | 150.000 | 150.000 | 150.000 |
| T.H (MWth) | **10091.92** | 10142.80 | 10116.87 | 10117.20 | 10214.90 | 10182.60 | 10104.73 | 10112.03 | 10144.24 | 10155.41 |
| T.Cost ($) | **44.937** | 35.166 | 37.802 | 43.253 | 10.000 | 10.791 | 42.434 | 45.701 | 48.404 | 32.459 |

**Appendix D**

**Table D.1.** Optimal CHPED schedule of the 7-unit system considering VPLE and PLs. (*P_d_* = 600 MW, and *H_d_* = 150 MWth).

| **Unit** | **MDBO** | **DBO** | **SCSO** | **AVOA** | **SCA** | **HHO** | **GWO** | **LLCA** | **ZOA** | **WOA** |
| --- | --- | --- | --- | --- | --- | --- | --- | --- | --- | --- |
| $P_{1}$ | 45.6 | 42.4 | 39.1 | 48.1 | 27.6 | 39.9 | 44.0 | 48.6 | 49.6 | 10.0 |
| $P_{2}$ | 98.5 | 98.5 | 20.4 | 98.5 | 26.6 | 98.3 | 98.7 | 98.8 | 98.3 | 46.8 |
| $P_{3}$ | 112.7 | 30.0 | 112.7 | 112.7 | 114.9 | 119.7 | 30.0 | 111.3 | 112.8 | 30.0 |
| $P_{4}$ | 209.8 | 294.7 | 294.9 | 209.8 | 297.7 | 209.8 | 294.8 | 208.8 | 209.8 | 380.1 |
| $P_{5}$ | 94.1 | 95.2 | 93.8 | 91.5 | 94.0 | 93.0 | 93.4 | 93.1 | 90.2 | 94.0 |
| $P_{6}$ | 40.0 | 40.0 | 40.0 | 40.0 | 40.0 | 40.0 | 40.0 | 40.1 | 40.0 | 40.0 |
| $H_{5}$ | 27.9 | 21.1 | 29.3 | 42.9 | 43.1 | 33.9 | 32.0 | 36.7 | 50.7 | 28.5 |
| $H_{6}$ | 75.0 | 75.0 | 73.7 | 69.8 | 65.0 | 69.4 | 72.6 | 69.5 | 51.5 | 74.0 |
| $H_{7}$ | 47.1 | 53.9 | 46.9 | 37.3 | 41.9 | 46.7 | 45.4 | 43.8 | 47.8 | 47.5 |
| $P_{losses}$ | 0.7 | 0.8 | 0.8 | 0.7 | 0.8 | 0.7 | 0.8 | 0.7 | 0.7 | 0.9 |
| T.P (MW) | 600.7 | 600.8 | 600.8 | 600.7 | 600.8 | 600.7 | 600.8 | 600.7 | 600.7 | 600.9 |
| T.H (MWth) | 150.0 | 150.0 | 150.0 | 150.0 | 150.0 | 150.0 | 150.0 | 150.0 | 150.0 | 150.0 |
| Total Cost ($) | 10094.23 | 10114.68 | 10118.28 | 10106.8 | 10216.55 | 10135.1 | 10115.68 | 10131.38 | 10135.34 | 10171.67 |

**Appendix E**

**Table E.1.** Optimal CHPED scheduling of the 7-unit system under VPLEs, PLs, and POZs (*P_d_* = 600 MW and *H_d_* = 150 MWth).

| **Unit** | **MDBO** | **DBO** | **SCSO** | **AVOA** | **SCA** | **HHO** | **GWO** | **LLCA** | **ZOA** | **WOA** |
| --- | --- | --- | --- | --- | --- | --- | --- | --- | --- | --- |
| $P_{1}$ | **44.047** | 36.230 | 35.101 | 36.691 | 17.149 | 39.165 | 40.774 | 16.625 | 17.129 | 11.633 |
| $P_{2}$ | **100.000** | 20.000 | 20.000 | 20.480 | 125.000 | 20.137 | 102.311 | 116.725 | 39.380 | 125.00 |
| $P_{3}$ | **112.674** | 30.000 | 30.011 | 30.997 | 30.000 | 30.093 | 112.734 | 39.587 | 115.741 | 30.125 |
| $P_{4}$ | **209.816** | 379.632 | 379.639 | 379.632 | 296.669 | 379.656 | 210.085 | 296.019 | 294.730 | 298.39 |
| $P_{5}$ | **94.204** | 95.052 | 96.165 | 93.114 | 92.041 | 91.864 | 94.836 | 91.816 | 93.801 | 95.545 |
| $P_{6}$ | **40.000** | 40.000 | 40.000 | 40.000 | 40.000 | 40.000 | 40.000 | 40.074 | 40.000 | 40.166 |
| $H_{5}$ | **27.062** | 22.066 | 15.527 | 33.476 | 45.676 | 40.849 | 23.758 | 43.388 | 29.435 | 19.166 |
| $H_{6}$ | **75.000** | 75.000 | 74.760 | 74.693 | 55.880 | 61.172 | 72.784 | 67.581 | 70.884 | 74.831 |
| $H_{7}$ | **47.938** | 52.934 | 59.713 | 41.831 | 48.444 | 47.979 | 53.458 | 39.031 | 49.681 | 56.003 |
| $P_{losses}$ | **0.740** | 0.914 | 0.915 | 0.913 | 0.859 | 0.914 | 0.740 | 0.846 | 0.781 | 0.861 |
| T.P (MW) | **600.74** | 600.914 | 600.915 | 600.913 | 600.859 | 600.914 | 600.740 | 600.846 | 600.781 | 600.86 |
| T.H (MWth) | **150.00** | 150.000 | 150.000 | 150.000 | 150.000 | 150.000 | 150.000 | 150.000 | 150.000 | 150.00 |
| T.Cost ($) | **10101.28** | 10146.48 | 10152.82 | 10154.36 | 10223.70 | 10165.70 | 10117.46 | 10219.20 | 10160.20 | 10181.12 |
